# MtTRC-1, a Novel Transcription Factor, Regulates Cellulase Production via Directly Modulating the Genes Expression of the *Mthac-1* and *Mtcbh-1* in *Myceliophthora thermophila*

**DOI:** 10.1128/aem.01263-22

**Published:** 2022-09-27

**Authors:** Nan Li, Yin Liu, Defei Liu, Dandan Liu, Chenyang Zhang, Liangcai Lin, Zhijian Zhu, Huiyan Li, Yujie Dai, Xingji Wang, Qian Liu, Chaoguang Tian

**Affiliations:** a College of Biotechnology, Tianjin University of Science & Technology, Tianjin, China; b Key Laboratory of Systems Microbial Biotechnology, Tianjin Institute of Industrial Biotechnology, Chinese Academy of Sciences, Tianjin, China; c National Technology Innovation Center of Synthetic Biology, Tianjin, China; d State Key Laboratory of Agrobiotechnology and MOA Key Laboratory of Soil Microbiology, College of Biological Sciences, China Agricultural University, Beijing, China; e Longda Biotechnology Inc, Shandong, Linyi, China; Kyoto University

**Keywords:** thermophilic fungi, *Myceliophthora thermophila*, cellulase production, transcription factor, MtTRC-1, MtHAC-1, *Mtcbh-1*, ER stress

## Abstract

The thermophilic fungus *Myceliophthora thermophila* has been used to produce industrial enzymes and biobased chemicals. In saprotrophic fungi, the mechanisms regulating cellulase production have been studied, which revealed the involvement of multiple transcription factors. However, in *M. thermophila*, the transcription factors influencing cellulase gene expression and secretion remain largely unknown. In this study, we identified and characterized a novel cellulase regulator (MtTRC-1) in *M. thermophila* through a combination of functional genomics and genetic analyses. Deletion of *Mttrc-1* resulted in significantly decreased cellulase production and activities. Transcriptome analysis revealed downregulation of not only the encoding genes of main cellulases but also the transcriptional regulator MtHAC-1 of UPR pathway after disruption of MtTRC-1 under cellulolytic induction conditions. Herein, we also characterized the ortholog of the yeast HAC1p in *M. thermophila*. We show that *Mthac-1* mRNA undergoes an endoplasmic reticulum (ER) stress-induced splicing by removing a 23-nucleotide (nt) intron. Notably, the protein secretion on cellulose was dramatically impaired by the deletion of MtHAC-1. Moreover, the colonial growth on various carbon sources was defective in the absence of MtHAC-1. Electrophoretic mobility shift assays and chromatin immunoprecipitation assays verified MtTRC-1 regulates the transcription of *Mthac-1* and the major cellulase gene *Mtcbh-1* by binding directly to the promoters in vitro and in vivo. Furthermore, DNase I footprinting assays identified the putative consensus binding site (5′-GNG/C-3′). These results revealed the importance of MtTRC-1 for positively regulating cellulase production. This finding has clarified the complex regulatory pathways involved in cellulolytic enzyme production.

**IMPORTANCE** In the present study, we characterized a novel regulator MtTRC-1 in *M. thermophila*, which regulated cellulase production through direct transcriptional regulation of the *Mthac-1* and *Mtcbh-1* genes. Our data demonstrated that MtHAC-1 is a key factor for the cellulase secretion capacity of *M. thermophila*. Our data indicate that this thermophilic fungus regulates cellulase production through a multilevels network, in which the protein secretory pathway is modulated by MtHAC-1-dependent UPR pathway and the cellulase gene expression is directly regulated in parallel by transcription factors. The conservation of *Mttrc1* in filamentous fungi suggests this mechanism may be exploited to engineer filamentous fungal cell factories capable of producing proteins on an industrial scale.

## INTRODUCTION

Plant biomass, consisting mainly of cellulose, hemicellulose, pectin, and lignin, represents an abundant and renewable energy source that can be used in biorefinery processes to produce valuable biofuels and biochemicals (1–3). The biological conversion of lignocellulosic biomass to various products requires plant cell wall-degrading enzymes (PCWDEs), which are commonly produced by filamentous ascomycetous fungi (4–6). There is interest in the utility of the thermophilic fungus *Myceliophthora thermophila* for biotechnological applications because of its ability to produce the complete set of thermostable carbohydrate-active enzymes (CAZymes) involved in biomass degradation (7–9). Previous research indicated that *M. thermophila* strain C1, which is an important platform for the industrial production of enzymes, can produce extracellular proteins, with yields of up to 100 g L^−1^ (10). More recently, *M. thermophila* was genetically manipulated to produce specific chemicals, such as malic acid (up to 180 g L^−1^), succinic acid, and fumaric acid (11, 12), via omics-based methods and versatile genetic techniques and tools, including high-efficiency transformation (13, 14) and CRISPR-Cas9/Cas12a systems (15, 16).

There are complex signaling networks involved in the gene expression associated with the production of lignocellulolytic enzymes in saprophytic fungi, including those related to environmental factors (e.g., light, nutrient availability, and pH), direct transcriptional regulation, and secretory pathway feedback (17–20). The transcriptional regulation of genes encoding cellulolytic enzymes contributing to plant biomass degradation is mainly dependent on transcription factors (21, 22). The key transcription factors, namely, CreA/CRE-1, CLR-1/A, CLR-2/B (ManR), and XYR1 (XLR-1/XInR), have been mostly studied in saprobic fungi, such as Neurospora crassa (23–27), Trichoderma reesei (28–34), and some Aspergillus spp. (35–42), which demonstrate they are the essential repressor or activators of most of the (hemi-)cellulolytic response. In N. crassa and Aspergillus spp., CreA/CRE-1 directly represses the expression of the mainly (hemi)-cellulases genes or some transcription factors involved in plant biomass degradation, while in *T. reesei* CRE1 functions as mainly “switch on/off” the transport of inducers/repressor depending on the environmental condition (reviewed in reference 22). In *T. reesei* and A. niger, XYR1/XlnR is the major transcriptional regulator of both hemicellulase and cellulase genes, while in N. crassa and A. nidulans, CLR-2/B modulates the expression of cellulolytic enzymes and XLR-1 regulates hemicellulose degradation (reviewed in reference 18).

In *M. thermophila*, MtXYR1 is the primary regulator of the expression of xylanolytic genes and genes involved in pentose transport and catabolism (13, 43). Similarly to its ortholog of XLR-1 in N. crassa, MtXYR1 is not essential for cellulose utilization and has no significant effects on the cellulases production (24, 43). In *M. thermophila*, the CRE-1 ortholog MtCRE1 is a key repressor of cellulase gene expression, and either the silencing or deletion of *Mtcre1* effectively improves cellulase production (15, 44). We previously characterized the new conserved Zn2Cys6 transcription factors NcCLR-4 and MtCLR-4 in N. crassa and *M. thermophila* and demonstrate that NcCLR-4/MtCLR-4 is a pivotal regulator for cellulolytic gene expression by directly regulating the expression of the essential transcriptional activators CLR-1 or CLR-2 and the key component of the cAMP signaling pathway adenylyl cyclase *cr-1* gene (45). Recently, we investigated the gene functions of the CLR-2 ortholog of MtCLR-2 in *M. thermophila* using the base editor and classical CRISPR/Cas9 systems (46). Our results show that the DNA-binding domain of MtCLR-2 is important for the fungal response to cellulose conditions and its fungus-specific motif is involved in fungal growth (46).

Because lignocellulases destined to be secreted to the cell exterior must be trafficked through the secretory system, the fungal intracellular secretory system (in particular components of the endoplasmic reticulum [ER]) also regulates the production of lignocellulolytic enzymes (17–19). The nascent PCWDE polypeptides generated during the cellulolytic response are translocated into the ER to be folded, modified, and assembled for secretion, which can lead to significant ER stress (47). This ER stress triggers the unfolded protein response (UPR) and ER-associated degradation (ERAD) to maintain protein homeostasis and cell survival or apoptosis (48, 49). The feedback mechanism, termed repression under secretion stress (RESS), helps to reduce ER load in filamentous fungi, which selectively downregulates the transcription of genes encoding extracellular enzymes under ER stress (50, 51). The mechanism underlying the UPR signaling pathway is highly conserved in fungi and has been thoroughly characterized in Saccharomyces cerevisiae (52), in which it is mediated by transmembrane proteinkinase with endoribonuclease activity Ire1p (inositol-requiring enzyme 1) and the downstream basic leucine zipper (bZIP) transcription factor Hac1p (homologous to HacA/HAC1 in filamentous fungi and X box-binding protein 1 [XBP-1] in mammals). During UPR induction, Ire1p cleaves the noncanonical intron of the gene encoding Hac1p to enable the production of an active transcription factor (i.e., the spliced mRNA of Hac1p), which translocates to the nucleus to upregulate the expression of its target genes; however, the length of the cleaved intron varies slightly among filamentous fungi (53–59). For example, *T. reesei hac1*, Aspergillus nidulans *hacA*, A. nidulans *hacA*, and Aspergillus fumigatus *hacA* contain a 20-nucleotide (nt) intron (53–55). In contrast, a 23-nt intron is cleaved from N. crassa *hac-1* in response to ER stress (56–58). In N. crassa, the expression of UPR-associated genes is reportedly upregulated during cellulose-related responses (56–58).

The regulatory mechanism of cellulase production as well as the ER stress-triggered UPR pathway in thermophilic fungi is still not completely understood. In this study, we identified a novel regulator of cellulase production, MtTRC-1 (Cys_2_His_2_ transcription factor, Mycth_90344), in the thermophilic fungus *M. thermophila*. The deletion of MtTRC-1 significantly decreased the cellulase production under crystalline cellulose (Avicel). We investigated the function of MtTRC-1 through gene disruption, transcriptome analysis, electrophoretic mobility shift assays (EMSAs), DNase I footprinting assays, and chromatin immunoprecipitation (ChIP) analysis. We show that MtTRC-1 positively regulates cellulase production by regulating the expression of a key regulator gene *Mthac-1* of the UPR pathway and the major cellulase gene *Mtcbh-1* through directly binding to their promoter region. We also demonstrate that the MtHAC-1 mRNA undergoes an ER stress-dependent splicing reaction by removing a 23-nt intron and plays a role in cellulase production, highlighting UPR pathway is a key factor for the cellulase secretion capacity in *M. thermophila*.

## RESULTS

### Identification of an *M. thermophila* Cys_2_His_2_ transcription factor that substantially affects cellulase production.

In order to discover the novel cellulase regulatory genes, we previously screened the cellulase secretion levels of 57 gene deletion strains of Neurospora crassa Cys_2_His_2_ zinc finger transcription factors by using 2% Avicel as the sole carbon source (60). We previously identified an N. crassa strain lacking the Cys_2_His_2_ transcription factor NCU10006 (FGSC 11396) (60), in which the cellulase production was significantly reduced in this deletion strain compared to the wild-type strain. The phylogenetic analysis of the NCU10006 orthologs showed broad conservation across filamentous fungi (Fig. S1A). This study aimed to examine the effects of its ortholog Mycth_90344 on cellulase production in the important thermophilic cellulolytic fungus *M. thermophila*. The expression profile analysis revealed transcript levels of the *Mycth_90344* gene were significantly upregulated when transferred to Avicel conditions and relatively higher in starch condition compared with the glucose condition (Fig. S1B).

**FIG 1:**
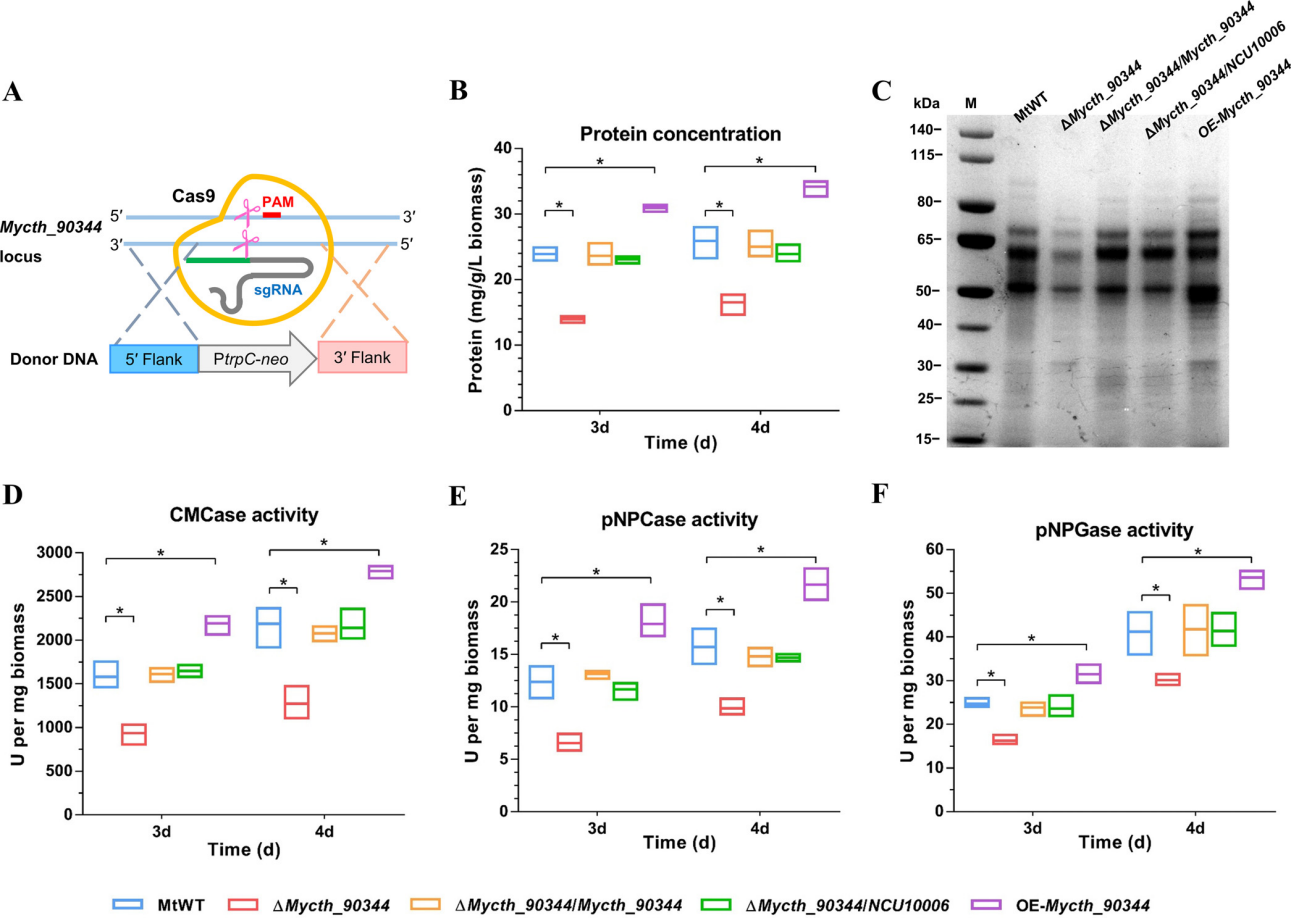
Protein production and enzyme activity phenotypes of *Myceliophthora thermophila* Δ*Mycth_90344* deletion mutants, complementation strains, and overexpression strains. (A) Schematic of the deletion of *Mycth_90344* in *M. thermophila* using the CRISPR-Cas9 system. (B) Protein concentrations of the culture supernatants for the *M. thermophila* strains grown for 4 days in 2% Avicel medium. (C) SDS-PAGE analysis of the proteins secreted by *M. thermophila* strains after 4 days on Avicel medium. (D to F) Activities of CMCase, pNPCase, and pNPGase in the culture supernatants for the *M. thermophila* strains. Bars marked by asterisks in each group differ significantly from the unmarked bars (Tukey’s HSD, *, *P* < 0.05). Error bars indicate the SD from three replicates. PAM, protospacer adjacent motif.

To investigate the function of the Mycth_90344 in *M. thermophila*, we created the deletion mutant Δ*Mycth_90344* by using our CRISPR/Cas9 system (Fig. 1A) (15). The deletion mutant was rescued via ectopic integration of the cassette of the *bar* marker and the full-length sequence of Mycth_90344 or NCU10006 with flanking regions. In addition, the encoding sequence of Mycth_90344 fused to the strong constitutive promoter P*tef-1* (translation elongation factor EF-1, MYCTH_2298136) was inserted ectopically into the chromosome of wild-type *M. thermophila* (MtWT) to form the Mycth_90344-overexpressing (OE) strain. The correct deletion and recombination events in the resulting mutants were confirmed via PCR and real-time quantitative PCR (RT-qPCR) analyses (Fig. S2).

The Δ*Mycth_90344* deletion mutant secreted approximately 41% to 42% less protein than the MtWT on crystalline cellulose (Avicel) (Fig. 1B). Additionally, compared with the corresponding activities of the MtWT strain, the CMCase, pNPCase, and pNPGase activities of the Δ*Mycth_90344* mutant decreased by approximately 27% to 47% (Fig. 1D to F). These results were confirmed by the SDS-PAGE protein profiling of the extracellular secretomes (Fig. 1C). The secreted protein amount and enzyme activities were restored to MtWT levels via native and interspecies complementation (Δ*Mycth_90344*/*Mycth_90344* and Δ*Mycth_90344*/NCU10006) (Fig. 1; Fig. S2). Moreover, an examination of the OE*-Mycth_90344* strain revealed significant increases in the amount of secreted protein and in cellulase activities (Fig. 1B to F). These findings suggested that Mycth_90344 is a regulator of cellulases production. Thus, it was named MtTRC-1 (i.e., *M. thermophila* transcriptional regulator of cellulases 1).

### Subcellular localization of MtTRC-1 in *M. thermophila*.

To determine the subcellular localization of MtTRC-1 under both glucose and cellulolytic growth conditions, the *Mttrc-1* open reading frame was fused to the *gfp* gene under the control of either the strong constitutive promoter (P*tef1*) or its native promoter region. The expression cassette was introduced into the deletion strain Δ*Mttrc-1* to generate the complementation strain expressing the MtTRC-1-GFP fusion protein. After a 16-h pregrowth period in minimal medium, the mycelia were cultured in both glucose and Avicel media for 24 h. The MtTRC-1-GFP signal was detected in the intracellular nuclei across the fungal hyphae under the control of either the P*tef1* or the native promoter; the signal overlapped with the blue fluorescence from mycelial nuclei stained with the nucleic acid-specific dye DAPI (Fig. S3). MtTRC-1 was exclusively located in the *M. thermophila* nucleus under either cellulose-inducing (Avicel) or noninducing (glucose) conditions.

### Transcriptome analysis of the MtTRC-1 deletion mutant.

**FIG 2:**
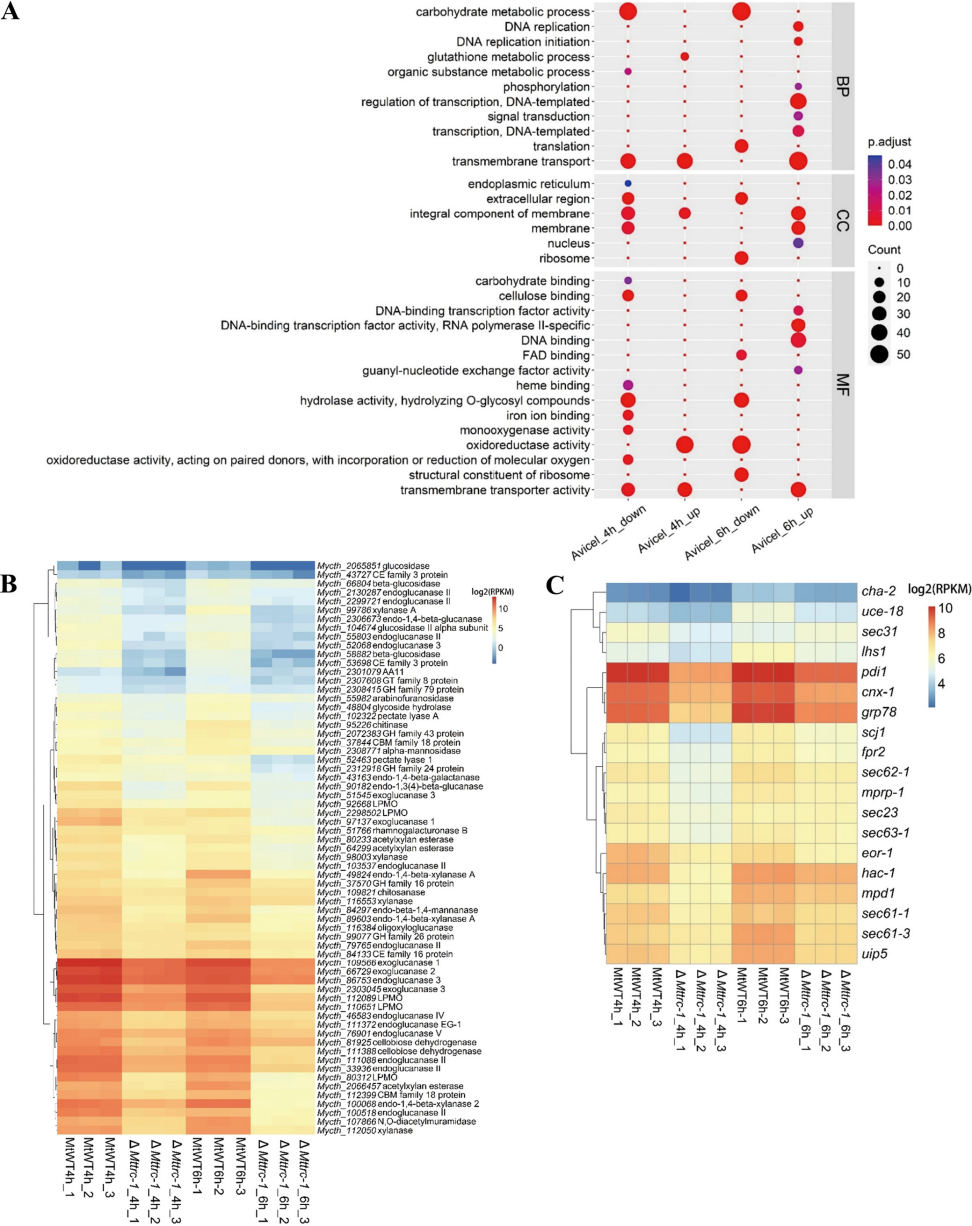
Comparative transcriptomics analysis of Δ*Mttrc-1* and MtWT grown in Avicel medium for 4 h and 6 h. (A) Gene Ontology analysis of the downregulated genes and upregulated genes differentially expressed between Δ*Mttrc-1* and MtWT on Avicel medium. (B) Heatmap analysis of expression profiles for the CAZy genes with statistically significant differences in transcript levels between Δ*Mttrc-1* versus MtWT under Avicel condition. Log-transformed expression values are color-coded. (C) Heatmap analysis of expression profiles for 19 genes associated with the secretory pathway in MtWT and Δ*Mttrc-1* strains under Avicel condition. Log-transformed expression values are color-coded.

Because of the observed decrease in the extracellular protein content due to a lack of MtTRC-1, the role of MtTRC-1 in cellulase production was investigated in more detail. On the basis of an RNA-seq analysis, we compared the transcriptional profiles of the MtWT and Δ*Mttrc-1* strains grown in Avicel medium for 4 h and 6 h, respectively (Table S2). Expression pattern analyses revealed that 918 and 1,649 genes were differentially expressed between Δ*Mttrc-1* and MtWT after 4 h and 6 h of Avicel induction, respectively. The transcription levels of 492 and 1,000 of these genes were significantly downregulated in Δ*Mttrc-1* under Avicel for 4 h and 6 h. The main enriched Gene Ontology (GO) terms among the downregulated genes were carbohydrate metabolic process, extracellular region, cellulose binding, and hydrolase activity (Fig. 2A; Table S3). Expression pattern analyses revealed that 426 and 649 genes were highly expressed in Δ*Mttrc-1* than MtWT under 4-h and 6-h Avicel, respectively. GO analysis showed these upregulated genes were overrepresented in transmembrane transport, DNA replication, regulation of transcription DNA-templated, integral component of membrane, DNA binding, transmembrane transporter activity, and oxidoreductase activity, including catalase-1, glutathione reductase, and cytochrome c peroxidase (Fig. 2A; Table S3).

Analyses of the transcriptional profiles indicated that 65 CAZyme genes involved in cellulose degradation were expressed at significantly lower levels in Δ*Mttrc-1* than in MtWT under both 4-h and 6-h Avicel (Fig. 2B). These genes included *Mycth_109566* (exoglucanase 1), *Mycth_66729* (exoglucanase 2), *Mycth_86753* (endoglucanase 3), *Mycth_66804* (beta-glucosidase), *Mycth_81925* (cellobiose dehydrogenase), *Mycth_80312* (lytic polysaccharide monooxygenases, LPMO), and *Mycth_110651* (LPMO). In addition to these CAZyme genes, the genes associated with the UPR signaling pathway also had lower expression levels in the Δ*Mttrc-1* mutant compared with the wild-type strain (Fig. 2C; Table S2). For example, the expression of the *Mthac-1* transcription factor gene (*Mycth_2310995*), which encodes the key regulator of the UPR pathway, was substantially downregulated in the Δ*Mttrc-1* mutant. This downregulated UPR genes also included genes encoding proteins involved in ER-mediated protein folding, such as *grp78* (*Mycth_2315513*), *pdi1* (*Mycth_2296005*), *mpd1* (*Mycth_2295433*), *cnx1* (*Mycth_2295005*), and *lhs1* (*Mycth_2312968*), and genes encoding proteins associated with protein translocation into the ER, including *sec61* translocon subunits (*Mycth_2314691* and *Mycth_2073127*), *sec62* (*Mycth_2310016*), and *sec63* (*Mycth_2303597*) (Fig. 2C).

We next examined the transcription of the genes encoding the major cellulases and the genes essential for the UPR pathway in the complementation and overexpression strains cultured on Avicel. The genes selected for the qPCR analysis included *cbh-1* (*Mycth_109566*), *cbh-2* (*Mycth_66729*), *gh5-1* (*Mycth_86753*), and *gh1-1* (*Mycth_115968*), two LPMO genes (*Mycth_80312* and *Mycth_110651*), and *Mthac-1*, *grp78*, *pdi1*, *cnx-1*, *lhs-1*, *prpA*, *ero-1*, and *fpr-2*. There were no obvious differences in the transcript levels of these genes between the complementation strains and the MtWT strain, whereas their transcript levels were significantly higher in the overexpression strains than in the MtWT strain (Fig. S2G). These observations were consistent with the increased secreted protein content and enzymatic activities in the overexpression strains.

To further investigate the role of MtTRC-1 in starch utilization, we next transcriptionally profiled the response of the MtWT and Δ*Mttrc-1* strains to the soluble starch for 4-h exposure. Under starch condition, the expression level of 337 genes was significantly downregulated in the Δ*Mttrc-1* mutant compared to MtWT, while the expression level of 363 genes increased in Δ*Mttrc-1* compared with MtWT (Table S2). GO enrichment analysis of these downregulated genes in Δ*Mttrc-1* uncovered three main categories, including carbohydrate metabolic process, transmembrane transport, and hydrolase activity and hydrolyzing O-glycosyl compounds (Fig. S4A; Table S3). These downregulated genes contained 39 CAZyme genes involved in starch degradation, including the key glucoamylase gene (*Mycth_72393*, *glaA*), an alpha-glucosidase gene (*Mycth_2303089*), *gh5-1* (*Mycth_86753*), and two LPMO genes (*Mycth_112089* and *Mycth_92668*) (Fig. S4B). The enriched GO categories of the upregulated genes contained oxidoreductase activity, transmembrane transport, and carbohydrate metabolic process (Fig. S4A; Table S3).

GO analysis between the MtWT and Δ*Mttrc-1* grown on Avicel and starch showed that 218 differentially expressed genes were significantly enriched in the 20 functional categories under Avicel condition, while only 125 differentially expressed genes were enriched in the 15 categories under starch condition (Fig. S4C). Comparing the 4-h Avicel and starch RNA-seq data between the wild-type and Δ*Mttrc-1* showed that only 66 genes were simultaneously downregulated and 18 genes were upregulated in the absence of *Mttrc-1* under both Avicel and starch conditions. These 66 downregulated genes contained 18 CAZyme genes, such as *cbh-2* (*Mycth_66729*), *gh5-1* (*Mycth_86753*), *Mycth_76901* (endoglucanase V), and three LPMO genes (*Mycth_92668*, *Mycth_112089*, and *Mycth_110651*) (Fig. S4D). This comparison indicates that MtTRC-1 has a more important role in the regulation of cellulose-degrading enzymes when cells are grown on Avicel than the upregulation of starch-degrading enzymes when grown on starch.

### MtHAC-1 plays a key role in the cellulase secretion.

**FIG 3:**
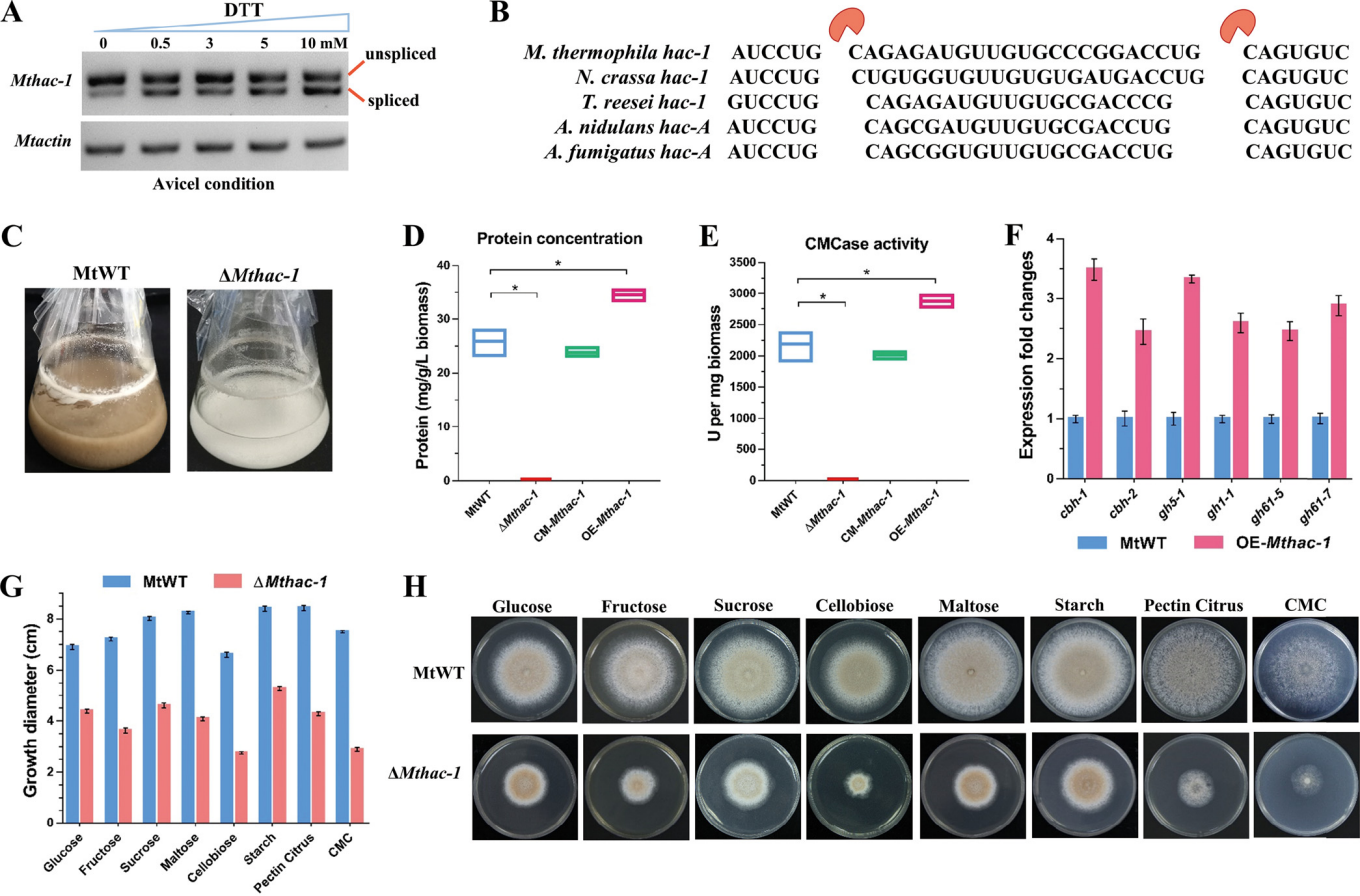
MtHAC-1 is a key regulator for cellulase secretion. (A) *Mthac-1* noncanonical splicing was monitored by qPCR. At 36-h postinoculation of Avicel medium, different concentrations of DTT were added to the MtWT cultures, which were then incubated for an additional 4 h. The spliced and unspliced *Mthac-1* were 172 and 195 bp long, respectively. The actin gene (*Mycth_2314852*) was used as a loading control. (B) Length comparison of the unconventionally spliced *hac-1* intron in different fungal species. (C) Growth of MtWT and Δ*Mthac-1* on Avicel medium after 4 days. The formation of tawny mycelia reflected the growth of the MtWT strain, whereas Δ*Mthac-1* growth was undetectable. (D to E) Assays for protein concentration and CMCase activity of the Δ*Mthac-1* and MtWT after 4 days culture in Avicel medium. (F) The transcription levels of major cellulase genes in the MtWT and OE-*Mthac-1* strains. Strains were pregrown in MM-glucose for 16 h, washed, and transferred to 2% Avicel medium for 4 h of incubation. (G to H) Growth comparison of MtWT and Δ*Mthac-1*. Plate growth diameter (G) and growth assay (H) on various carbon sources (1% wt/vol). Cultures were incubated at 45°C for 4 days. All assays were performed with multiple culture replicates.

Previous studies on N. crassa demonstrated that the transcription factor HAC-1 is activated by unconventional mRNA splicing for growth on cellulose, suggesting the UPR pathway is triggered when N. crassa degrades cellulose (56–58). To determine how the HAC-1-dependent UPR pathway of *M. thermophila* is modulated by ER stress, fungal cultures were treated with different concentrations of dithiothreitol (DTT) for 4 h while being grown on cellulose. According to the gel electrophoresis results, the growth on cellulose for 36 h without DTT initially triggered the splicing of the *Mthac-1* (Mycth_2310995) precursor mRNA (Fig. 3A). This splicing was induced in response to 0.5 mM DTT and highly induced at 4 h after the 10 mM DTT treatment. The sequencing of the *Mthac-1*-amplified products (Fig. 3A) and the subsequent comparison with the corresponding sequences in different fungal species detected the removal of a 23-nt inhibitory intron fragment from *Mthac-1* mRNA under ER stress conditions (Fig. 3B). The *M. thermophila Mthac-1* mRNA contained an intron (23 nt), which was similar in size to the N. crassa *hac-1* intron (23 nt), and slightly longer than the introns in other filamentous fungi, such as *T. reesei* (20 nt), A. nidulans (20 nt), and A. fumigatus (20 nt) (50–55).

To explore how the MtHAC-1-mediated UPR pathway affects cellulase production, we generated the Δ*Mthac-1* deletion mutant, complementation strain (CM-*Mthac-1*) and the *Mthac-1* overexpressing strain (OE-*Mthac-1*). The correct recombination events in the resulting mutants were confirmed via PCR and RT-qPCR analyses (Fig. S5A to E). The Δ*Mthac-1* mutant grew very poorly and failed to germinate in liquid shake flasks when 2% (wt/vol) crystalline cellulose was used as the sole carbon source (Fig. 3C). In contrast to MtWT, the Δ*Mthac-1* mutant lacked detectable secreted proteins and cellulase activity under cellulolytic conditions (Fig. 3D and E). These observations were in accordance with the results of previous studies on the N. crassa Δ*hac-1* mutant (57, 58). The protein content and cellulase activity of the complementation strain CM-*Mthac-1* reverted to the same level as those of wild-type strain MtWT, while the *Mthac-1* overexpressing strain OE-*Mthac-1* showed higher protein level and cellulase activity than MtWT (Fig. 3D and E). The expression levels of six major cellulase encoding genes were enhanced in the OE-*Mthac-1* strain compared with the parental strain MtWT under 4-h Avicel induction (Fig. 3F). To test the role of MtHAC-1 on utilization of other carbohydrates in *M. thermophila*, we assessed the growth of the Δ*Mthac-1* mutant on various carbon sources. Compared with the MtWT strain, the Δ*Mthac-1* mutant exhibited significantly reduced growth on the all tested carbon sources (Fig. 3G and H). The severe growth defect on sodium carboxymethyl cellulose (CMC) was consistent with the poor growth phenotype observed in the Δ*Mthac-1* mutant on the liquid medium containing crystalline cellulose as the sole carbon source. When using the soluble inducer cellobiose as the sole carbon source, deletion of *Mthac-1* had less defect growth in liquid shake flasks compared with the Avicel condition (Fig. S5F). The Δ*Mthac-1* deletion mutant also showed greatly reduced protein concentration and cellulase activities compared with the MtWT under cellobiose condition (Fig. S5G).

### MtTRC-1 positively regulates cellulase production through the HAC-1-dependent UPR pathway.

**FIG 4:**
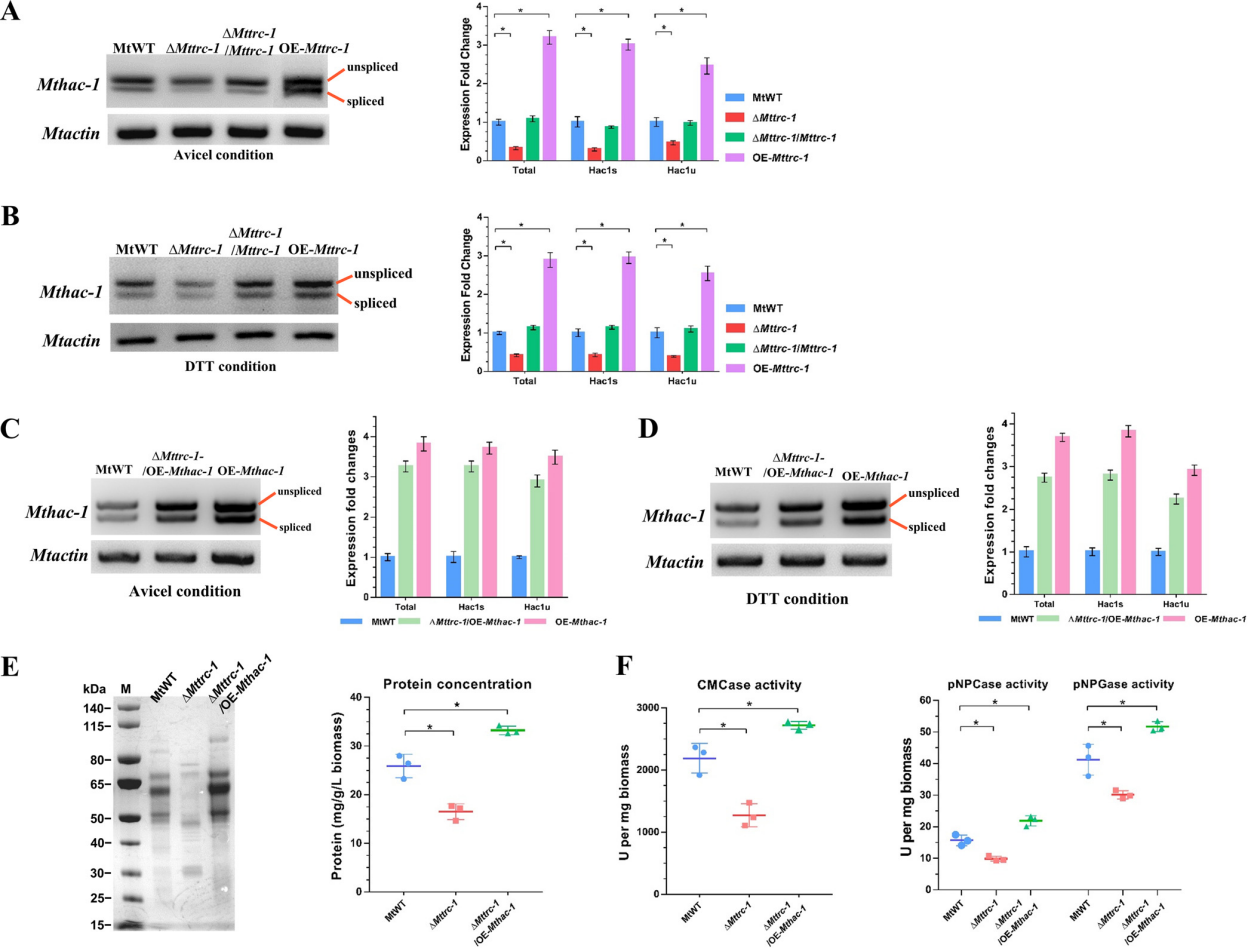
MtTRC-1 regulates cellulase production through the MtHAC-1-dependent UPR signaling pathway. (A to B) Deletion of MtTRC-1 resulted in a significant decrease in the transcription of *Mthac-1*. Fungal strains were pregrown on glucose medium for 16 h and then treated with Avicel (A) or 10 mM DTT (B) for 4 h. The gel electrophoresis results (left) and the expression level fold changes (right) determined by qPCR were used to reveal changes in *Mthac-1* transcription. (C to D) The transcript abundance of the spliced and unspliced *Mthac-1* in the MtWT, OE-*Mthac-1* and Δ*Mttrc-1*/*Mthac1*-OE strains under Avicel (C) and DTT (D) conditions for 4 h. (E to F) Overexpressing *Mthac-1* restored the secretion of extracellular proteins according to the SDS-PAGE analysis and the production level (E) and enzyme activities (F) in Δ*Mttrc-1* on Avicel medium for 4 days. *, *P* < 0.05. Error bars represent the SD from three replicates.

Our transcriptome analysis showed that the transcription levels of *Mthac-1* and other UPR signaling cascade genes were significantly lower in the Δ*Mttrc-1* mutant than in the MtWT control when the strains were grown on Avicel (Fig. 2C; Table S2). We analyzed the expression levels of the spliced and unspliced *Mthac-1* in the *Mttrc-1* deletion, complementation, and overexpression strains and the MtWT strain grown in the presence of Avicel. As expected, the spliced and unspliced *Mthac-1* transcript levels decreased significantly in the Δ*Mttrc-1* mutant, whereas the transcript levels were rescued in the complementation strain and were obviously increased in the overexpression strain (relative to the transcript levels in MtWT) (Fig. 4A). We also examined the expression levels of the spliced and unspliced *Mthac-1* under artificial ER-stress conditions (4-h treatment with 10 mM DTT) (Fig. 4B). When induced by DTT, the expression of the spliced and unspliced *Mthac-1* was also lower in the Δ*Mttrc-1* mutant compared with the MtWT strain (Fig. 4B). The transcript abundance of the spliced and unspliced *Mthac-1* were significantly increased in the *Mthac-1* overexpressing strains OE-*Mthac-1* and Δ*Mttrc-1*/*Mthac1*-OE, compared with the MtWT strain on both Avicel and DTT after 4 h (Fig. 4C and D). These data suggest MtTRC-1 is an important factor for the regulation of *Mthac-1* expression in response to cellulose and ER stress conditions.

### Constitutive expression of MtHAC-1 rescues the defective cellulase production resulting from *Mttrc-1* deletion.

Because the lack of MtTRC-1 adversely affected *Mthac-1* transcription in strains grown on Avicel, we hypothesized that downregulated *Mthac-1* expression may partially account for the decreased cellulase production in the Δ*Mttrc-1* mutant. To test this hypothesis, *Mthac-1* was expressed in the Δ*Mttrc-1* mutant by introducing the *Mthac-1* overexpression cassette, which was under the control of the constitutive *tef-1* promoter. The overexpression of *Mthac-1* restored the extracellular protein production and enzyme activities of the generated Δ*Mttrc-1*/*Mthac1*-OE strain grown on Avicel (Fig. 4E and F). In fact, cellulase secretion and activity increased in the Δ*Mttrc-1*/*Mthac1*-OE strain, implying that the UPR pathway plays a key role in the production of cellulases in *M. thermophila*.

### MtTRC-1 affects the intracellular reactive oxygen species level.

There is accumulating evidence of the ER stress-induced production of reactive oxygen species (ROS) during the ER-mediated protein folding process and the relationship among ER stress, ROS, and redox signaling mediators such as PDI-1 and ERO-1 (61). We compared the ROS levels in the MtWT, Δ*Mttrc-1*, and Δ*Mttrc-1*/*Mttrc-1* strains and clarified the effects of a treatment with 1 mM DTT for 0.5 h and 1 h (i.e., ER stress conditions) on ROS accumulation. The ROS levels in fungal mycelia exposed to DTT were determined on the basis of 2′,7′-dichlorodihydrofluorescein diacetate (DCFH-DA) staining. The intracellular ROS level was higher in the Δ*Mttrc-1* mutant than in the MtWT strain, whereas there was no difference in the ROS contents of the Δ*Mttrc-1*/*Mttrc-1* mutant and the MtWT strain (Fig. S2H). The results were consistent with the upregulated expression of the genes encoding enzymes associated with oxidoreductase activity and oxidative stress response, including catalase-1, glutathione reductase, and cytochrome c peroxidase, in the Δ*Mttrc-1* mutant (Table S2).

### MtTRC-1 binds directly to the *Mthac-1* promoter region *in vitro* and *in vivo*.

**FIG 5:**
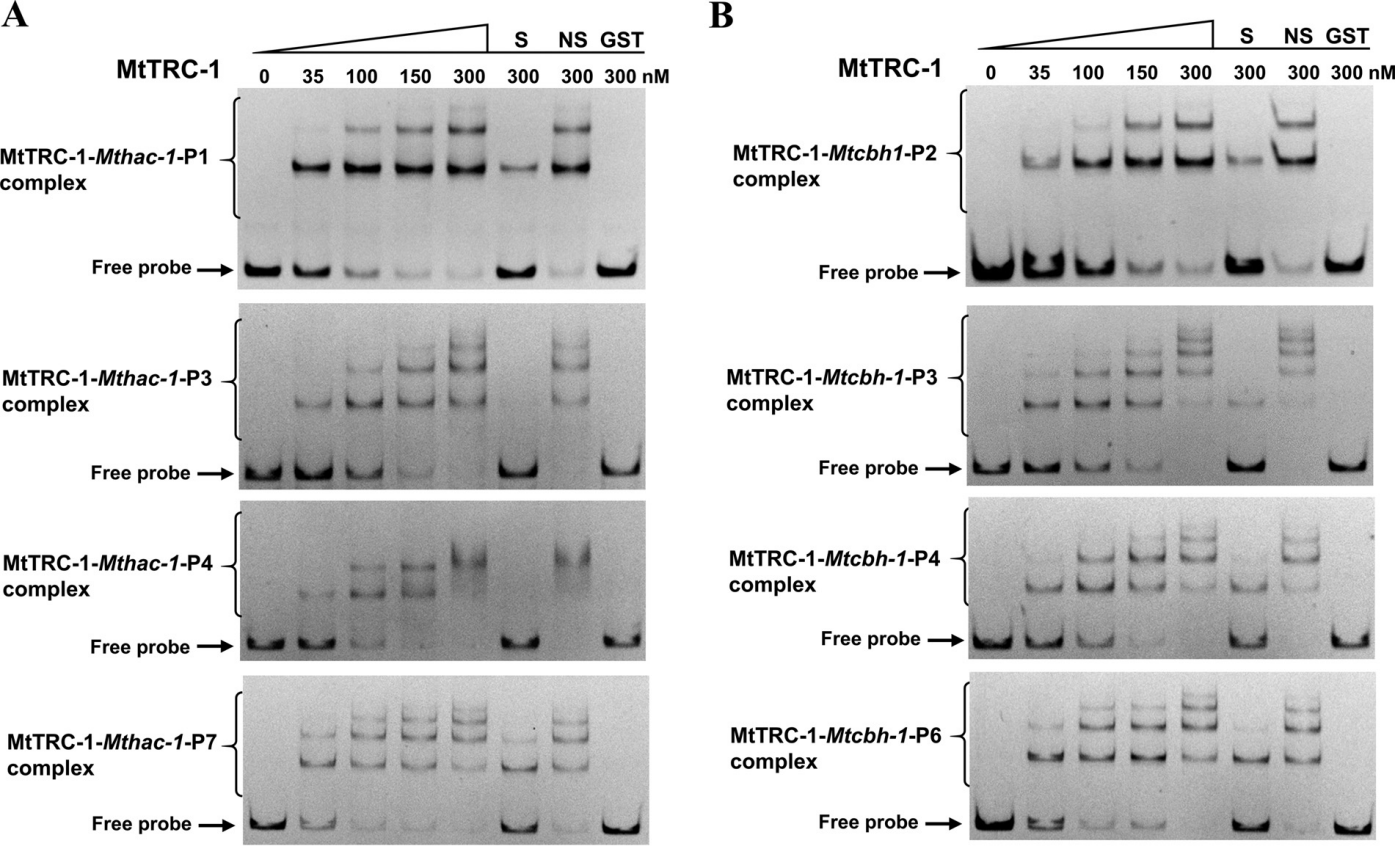
Electrophoretic mobility shift assays (EMSAs) for MtTRC-1. (A) EMSAs of the binding of MtTRC-1 to the upstream region of *Mthac-1*. (B) EMSAs of the interaction between MtTRC-1 and the *Mtcbh-1* promoter region. Each lane contained 10 ng Cy5-labeled probe and the indicated amounts of the purified MtTRC-1 DNA-binding domain (nM). The shifts in (A) and (B) were verified to be specific by adding 100-fold excess of unlabeled specific (S) and nonspecific (NS) competitor DNA. Purified GST was used as a negative control to exclude nonspecific binding.

To assess whether MtTRC-1 can directly regulate *Mthac-1* expression, EMSAs and ChIP-qPCR assays were performed. The EMSAs involved the *Mthac-1* promoter region and the DNA-binding domain (DBD) of MtTRC-1. Specifically, the MtTRC-1 DBD was fused to the C-terminus of a GST tag to generate the GST-MtTRC-1-DBD fusion protein, which was expressed in Escherichia coli cells and then purified (Fig. S6A). Seven Cy5-labeled probes covering the *Mthac-1* promoter region (nucleotide positions: −1 to −1,898) were amplified by PCR and assessed regarding their ability to bind to GST-MtTRC-1-DBD. In the EMSAs, the recombinant MtTRC-1 bound to the *Mthac-1* promoter region in a typical protein concentration-dependent manner (Fig. 5A; Fig. S7A). Retardation was observed after the addition of 10 nM GST-MtTRC-1-DBD. Additionally, GST-MtTRC-1-DBD had a high affinity for the Cy5-labeled PCR fragments of the *Mthac-1*-P1, *Mthac-1*-P3, *Mthac-1*-P4, and *Mthac-1*-P7 promoter regions. The shifts were verified to be specific by adding 100-fold excess of unlabeled specific (S) and nonspecific (NS) competitor DNA. Purified GST (300 nM) was used as a negative control to exclude nonspecific binding.

**FIG 6:**
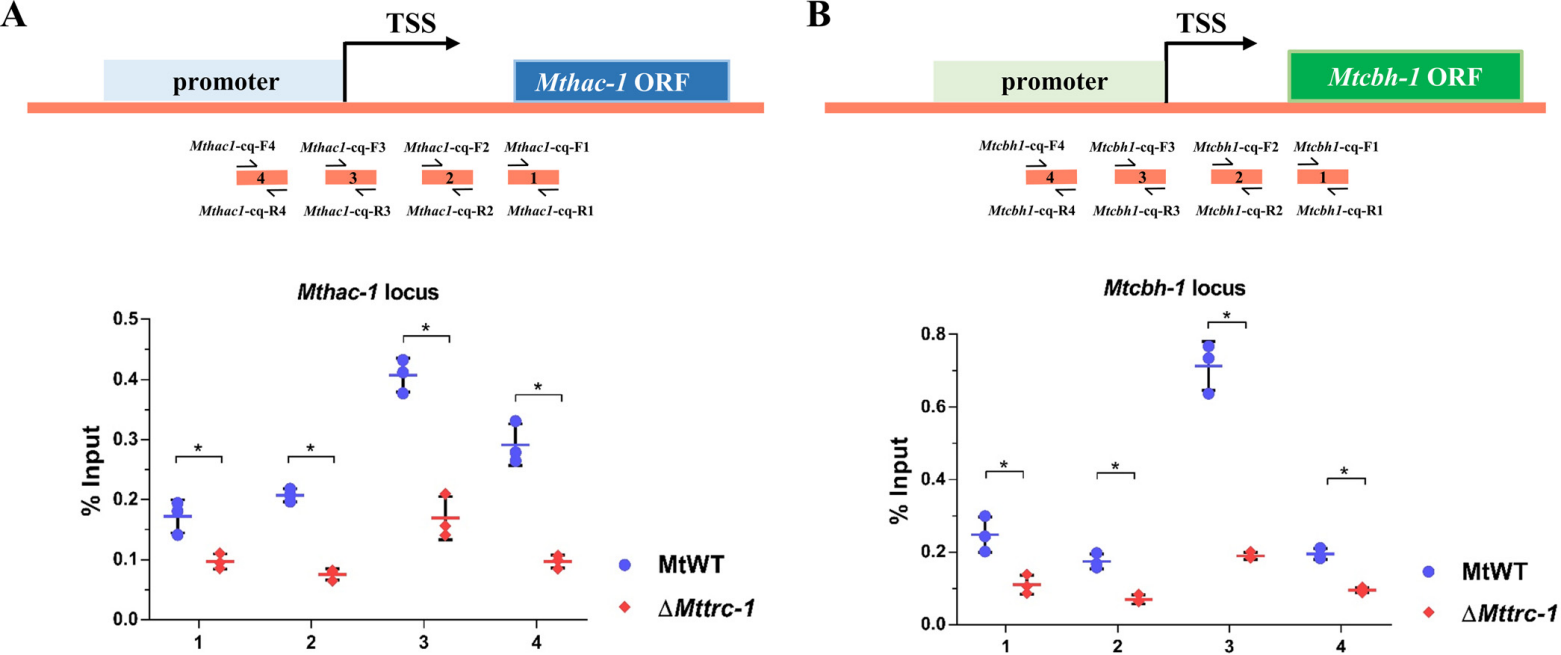
ChIP-qPCR assays showing the extent of the binding of MtTRC-1 at the *Mthac-1* locus (A) and the *Mtcbh-1* locus (B) in MtWT and Δ*Mttrc-1* after a 4-h induction on 2% Avicel medium. Short black lines (primer pairs 1 to 4) under the schematic diagram of the *Mthac-1* and *Mtcbh-1* genes indicate the regions detected by ChIP-qPCR. TSS, transcription start site; ORF, open reading frame. Error bars represent the SD from three replicates. Significance was assessed by Tukey’s HSD (*, *P* < 0.05).

To investigate whether MtTRC-1 regulates the expression of *Mthac-1 in vivo*, we generated an anti-MtTRC-1 antibody, which recognized a specific protein fragment (with the predicted molecular weight) in the MtWT and *Mttrc-1*-*gfp* overexpression strains, but not in the Δ*Mttrc-1* mutant (Fig. S6B). This antibody was also used in the ChIP-qPCR assays, which revealed MtTRC-1 was highly enriched at the *Mthac-1* locus, especially at the promoter (primer pair 4) and transcription start site region (primer pair 3), in the MtWT strain, but not in the Δ*Mttrc-1* mutant (Fig. 6A). These EMSA and ChIP-qPCR results demonstrated that MtTRC-1 regulates *Mthac-1* expression by binding directly to the promoter region *in vitro* and *in vivo*.

### MtTRC-1 can regulate cellulase gene expression by binding directly to the *Mtcbh-1* promoter region.

The transcriptome data indicated that the expression levels of many CAZyme genes, including the major cellulase gene *Mtcbh-1* (Mycth_109566), were significantly downregulated in the Δ*Mttrc-1* mutant. What caused the *Mtcbh1* downregulation at transcriptional level? Thus, we subsequently focused on clarifying the factor responsible for the downregulation of *Mtcbh-1* at the transcriptional level. Neither of the main transcriptional activators (CLR-1 and CLR-2) were obviously downregulated in the Δ*Mttrc-1* mutant. We speculated that MtTRC-1 may regulate cellulase gene expression directly. Therefore, we tested whether MtTRC-1 can bind directly to the *Mtcbh-1* promoter region, like other classic cellulase regulators. We then investigated the regulatory effects of MtTRC-1 on *Mtcbh-1* transcription by performing EMSAs and ChIP-qPCR assays. The DNA fragments were labeled with Cy5 via PCR amplification and used for EMSAs. Seven Cy5-labeled probes covering the *Mtcbh-1* promoter region (nucleotide positions: −1 to −1,843) were generated for analyses of their binding to MtTRC-1 (Fig. 5B; Fig. S7B). The EMSA results confirmed GST-MtTRC-1-DBD can bind to the Cy5-labeled *Mtcbh-1*-P2/P3/P4/P6 promoter fragments in a concentration-dependent manner (Fig. 5B). These shifts were specific, as a 100-fold excess of unlabeled specific (S) probes competed for binding (Fig. 5B; Fig. S7B). Nonspecific (NS) competitor probes and the GST were used as a negative controls. The ChIP-qPCR data reflected the substantial enrichment of MtTRC-1 at the *Mtcbh-1* locus (Fig. 6B). The deletion of *Mttrc-1* resulted in a considerable decrease in the enrichment of MtTRC-1 at the *Mtcbh-1* locus, especially in the promoter region (primer pair 3) (Fig. 6B). Interestingly, the EMSA and ChIP-qPCR assay results suggested that MtTRC-1 also regulates cellulase gene expression by binding directly to the corresponding promoter regions *in vitro* and *in vivo*.

Taken together, these results imply MtTRC-1 modulates cellulase production through regulating the major cellulase gene *Mtcbh-1* expression.

### Identification of the precise MtTRC-1-binding sites in the upstream regions of *Mthac-1* and *Mtcbh-1*.

To precisely locate the *Mthac-1* and *Mtcbh-1* promoter regions that MtTRC-1 binds to, DNase I footprinting assays were performed using purified GST-MtTRC-1-DBD and the FAM-labeled promoter fragments *Mthac-1*-P7-FP and *Mtcbh-1*-P4-FP, which produced the strongest specific shifts (Fig. 5).

**FIG 7:**
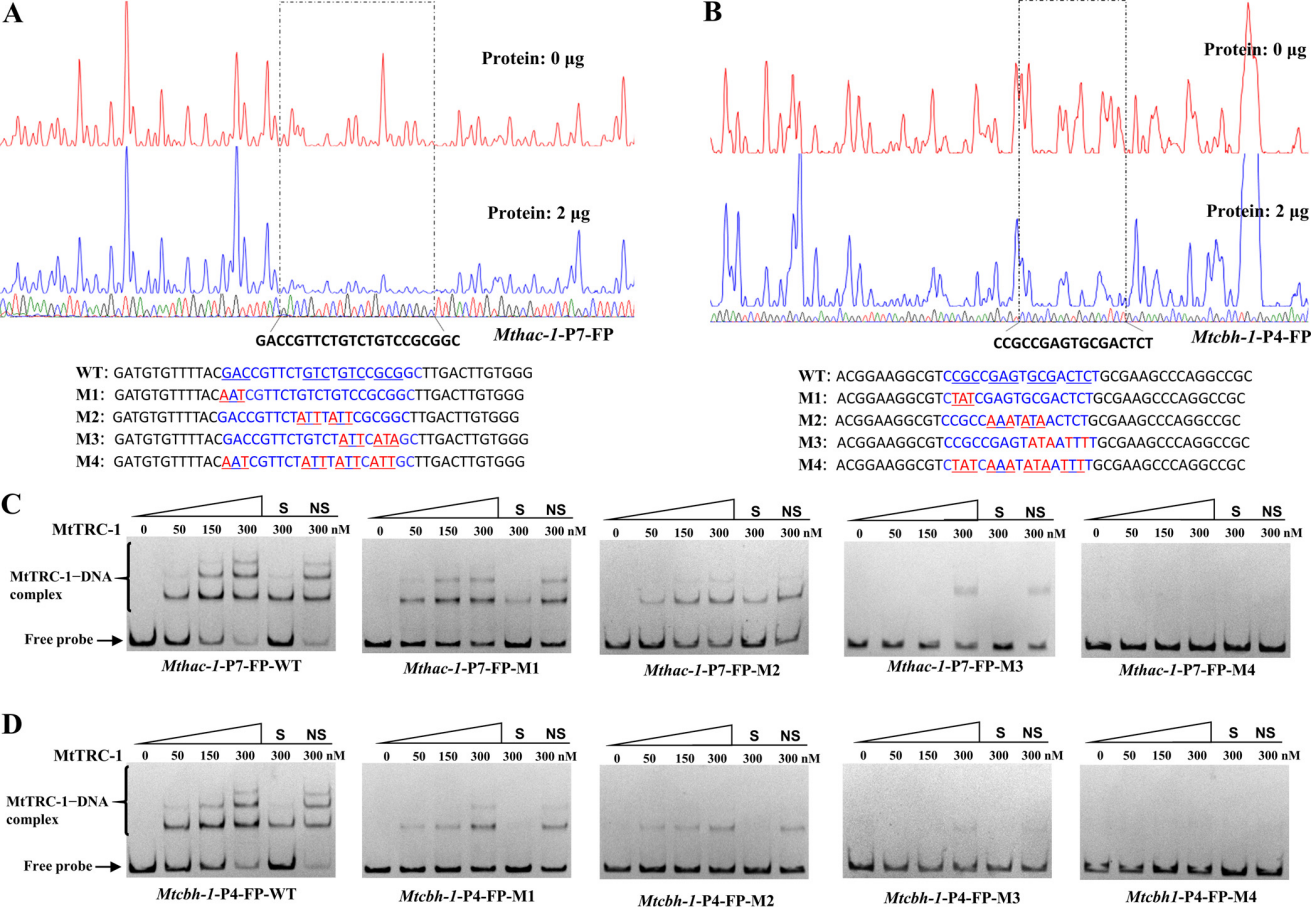
Identification of the MtTRC-1-binding site in the *Mthac-1*-P7 and *Mtcbh-1*-P7 upstream regions. (A and B) Identification of the MtTRC-1-protected *cis-*elements in the upstream regions of *Mthac-1* (*Mthac-1*-P7-FP) (A) and *Mtcbh-1* (*Mtcbh-1*-P4-FP) (B) on the basis of the DNase I footprinting assay results. The upper two colored lines represent the different amounts of MtTRC-1 used (red, 0 μg; blue, 2 μg). The lower line corresponds to the sequencing map of the *Mthac-1* (A) and *Mtcbh-1* (B) promoter regions. The DNA sequence protected from DNase I (dashed box) is presented. The sense strand sequences of *Mthac-1*-P7-FP-WT and *Mtcbh-1*-P4-FP-WT and their four mutated fragments (M1 to M4) are shown (5′ to 3′). The putative MtTRC-1-binding regions are highlighted in blue. The lines under the sequences indicate the bases in the mutated fragments (highlighted in red) that differ from the 5′-GNG/C-3′ sequence in the WT probes. (C to D) Analyses of the binding of MtTRC-1 to the WT and four mutated probes of *Mthac-1*-P7-FP (C) and *Mtcbh-1*-P4-FP (D). Different amounts of protein with a constant amount (10 ng) of Cy5-labeled DNA probe were added to each reaction mixture. The shifts were verified to be specific by adding 100-fold excess of unlabeled specific (S) and nonspecific (NS) competitor DNA. The presented results are from three experiments that produced similar results.

Along with the increased abundance of MtTRC-1, one apparently protected region was detected in *Mthac-1*-P7-FP, with four highly protected GNC/G motifs (Fig. 7A). A previous study involving protein binding microarrays indicated that C_2_H_2_ zinc finger transcription factors prefer GNN subsites (62). Accordingly, to verify these motifs are involved in the binding of MtTRC-1, the MtTRC-1-protected region of *Mthac-1*-P7-FP was mutated via site-directed mutagenesis. Four Cy5-labeled fragments (M1 to M4) containing mutations were generated for EMSAs (Fig. 7A). The mutations in the M1 probe drastically decreased the binding affinities, while the mutations in the M2 and M3 probes exhibited stronger effect on binding profiles (Fig. 7C). Binding was undetectable for the M4 probe, in which all GNC motifs were mutated, indicating that the combined GNC motifs are critical for the binding of MtTRC-1 to the *Mthac-1* promoter.

The DNase I footprinting assay was also performed using the FAM-labeled *Mtcbh-1*-P4-FP probe to determine the MtTRC-1-binding site in *Mtcbh-1*. A protected DNA region with high affinity for MtTRC-1 was identified in *Mtcbh-1*-P4-FP (Fig. 7B). Four GNG motifs were detected in this MtTRC-1-protected region. We mutated the four GNG motifs and then examined the effects of these mutations on binding affinities on the basis of EMSAs. Each Cy5-labeled mutated probe significantly decreased MtTRC-1 binding activities, with mutations to four GNG motifs leading to a complete lack of binding (Fig. 7D). These results reflected the importance of the combined GNG/C motifs for the binding of MtTRC-1 to the *Mthac-1* and *Mtcbh-1* promoters.

**FIG 8:**
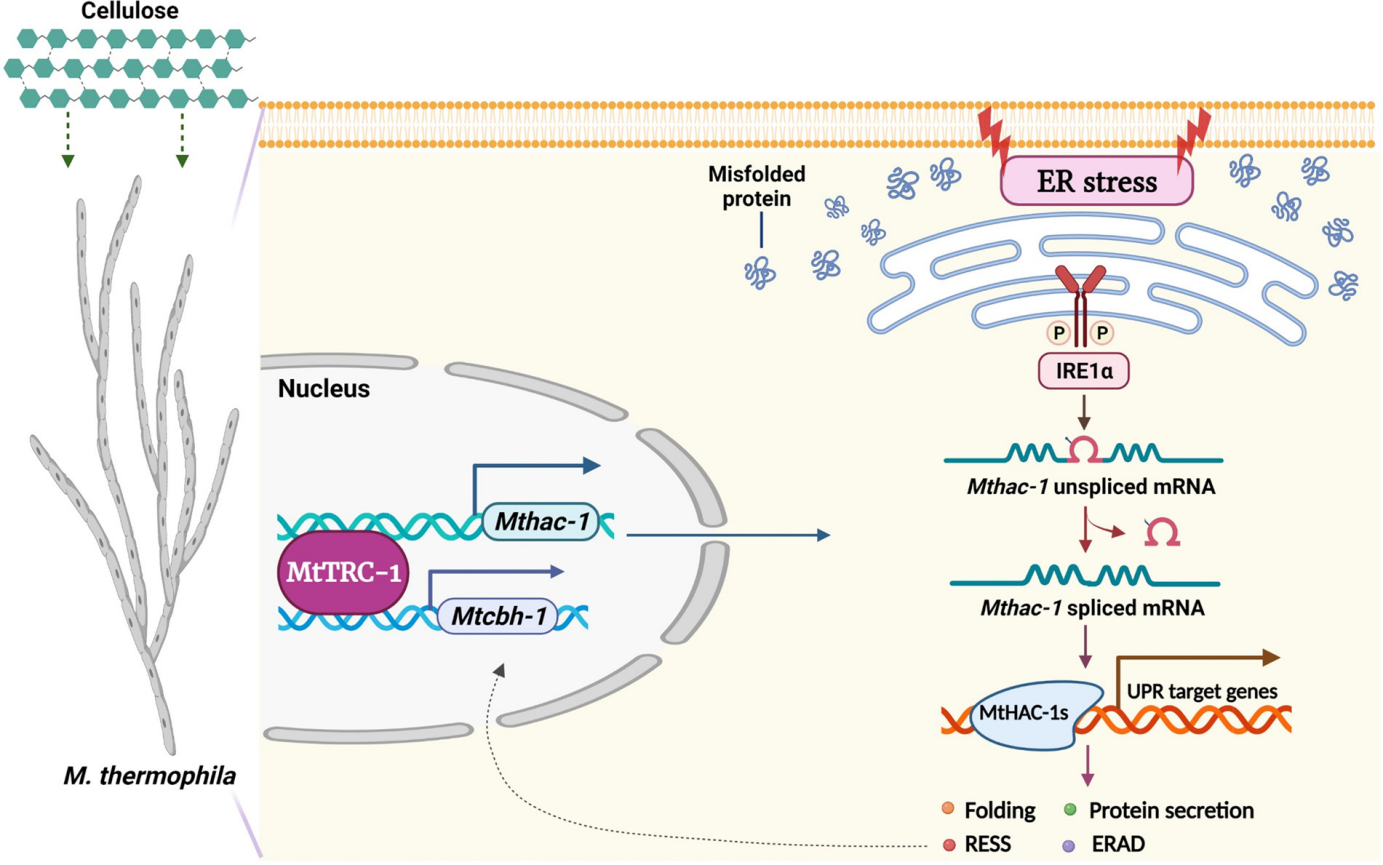
Schematic model depicting the regulatory role of MtTRC-1 during *M. thermophila* cellulase production. The transcript of the key UPR regulator MtHAC-1 has a 23-nt intron removed during ER stress-induced splicing. MtHAC-1 plays an important role in the cellulase secretion pathway in fungi grown in the presence of cellulose. Specifically, MtTRC-1 directly regulates the expression level of *Mtcbh-1* and *Mthac-1* by binding to their promoter regions *in vitro* and *in vivo*. Thus, this mechanism can precisely regulate cellulase production by two level network involving the MtHAC-1 mediated UPR signaling, as well as the transcriptional regulation of the major cellulase genes in parallel.

The EMSA, ChIP, and DNase I footprinting data indicated that MtTRC-1 participates in the regulation of cellulase production through direct transcriptional regulation of *Mthac-1* and *Mtcbh-1* in *M. thermophila* (Fig. 8).

## DISCUSSION

Filamentous fungi are important for the biotechnological production of industrial enzymes and chemical products (4–6). Thus, studies on either transcriptional regulation or protein secretion are critical for characterizing and constructing complex expression systems involving filamentous fungi (63, 64). Lignocellulolytic enzyme production in saprophytic filamentous fungi is controlled at the transcriptional level via the activities of regulators, such as CreA/CRE-1 (23, 28, 29), CLR-1/A, CLR-2/B (25–27), and XYR-1/XLR-1/XInR (24, 31–33), or at the posttranslational level through the HAC-1 mediated UPR signaling pathway (17, 18, 47). Although there is increasing research on the regulation of the lignocellulolytic response in saprotrophic fungi, the associated regulatory mechanisms in *M. thermophila* are largely unknown. Recent advances in research involving genomic techniques, multiomics data, and synthetic biology approaches have begun to elucidate how the thermophilic fungus *M. thermophila* degrades plant biomass, which may have implications for biotechnological applications (7–12).

In this study, we identified and characterized a novel transcriptional regulator (MtTRC-1) that positively mediating cellulase production through directly regulating the expression levels of *Mtcbh-1* and *Mthac-1* in *M. thermophila* by combining functional genomic assays and molecular genetic analyses. The deletion of the nuclear-localized regulator MtTRC-1 dramatically decreased cellulase production, whereas the overexpression of MtTRC-1 increased cellulase secretion in fungi grown on cellulose (Fig. 1; Fig. S2). We further clarified the molecular mechanism underlying the MtTRC-1-mediated regulation of cellulase production. Our initial transcriptome and qPCR analyses indicated that *Mthac-1* expression was significantly lower in the Δ*MtTrc-1* mutant than in MtWT in response to Avicel and artificial ER stress (Fig. 2 and 4). Thus, MtTRC-1 may affect the UPR signaling pathway through the transcription factor MtHAC-1.

Earlier research identified HAC-1/HAC-A as the key transcriptional activator of the UPR pathway (48–54). Fungal UPR pathways are induced under ER stress conditions, which may be relevant for overcoming the bottlenecks associated with the industrial production of proteins (17–19, 47). Additionally, HAC-1/HAC-A gene expression is upregulated in N. crassa in response to crystalline cellulose (56) as well as in *T. reesei* and Aspergillus species during the production of secreted enzymes (53, 54, 59). The deletion of either IRE-1 or HAC-1 reportedly leads to a significant decrease in cellulase secretion in N. crassa grown on Avicel (57, 58). However, HAC-1 homologs in thermophilic fungi have not been functionally characterized. Hence, we conducted this study to clarify the role of MtHAC-1 in *M. thermophila*. We first monitored the noncanonical splicing of *Mthac-1* under ER stress conditions (DTT treatment) and generated the Δ*Mthac-1 M. thermophila* knockout mutant (Fig. 3). During the ER stress-dependent splicing, a 23-nt intron was removed from *Mthac-1* mRNA, which is consistent with the size of the intron removed during the splicing of homologs in filamentous fungi (approximately 20 nt) (53–58), including N. crassa (23 nt) (56–58). As expected, the deletion of MtHAC-1 in the Δ*Mthac-1* mutant resulted in severely defective growth on Avicel and no detectable protein secretion and cellulase activities (Fig. 4). These findings are in accordance with the N. crassa phenotypes in earlier studies (57, 58). The overexpression of *Mthac-1* in the Δ*Mttrc-1* mutant restored the cellulase secretion and enzyme activities (Fig. 4). Thus, MtHAC-1-dependent UPR signaling is important for the lignocellulase production in *M. thermophila*. Furthermore, EMSAs and DNase I footprinting assays combined with ChIP-qRCR analyses demonstrated that MtTRC-1 directly regulates *Mthac-1* expression by binding to the promoter region *in vitro* and *in vivo* (Fig. 5–7). Considered together, these findings indicate that MtTRC-1 regulates cellulase production via the MtHAC-1-mediated protein secretory pathway (Fig. 8).

In ascomycetous filamentous fungi, the expression of (hemi-)cellulase genes is commonly directly regulated by various transcription factors (18, 21–24). However, to date, only a few transcription factors, including MtXlr-1 (13, 43), MtCRE-1 (44), and MtCLR-4 (45), and MtCLR-2 (46), have been confirmed to participate in the direct transcriptional regulation of (hemi-)cellulase genes in *M. thermophila*. Our transcriptome data reflected the significantly downregulated expression of cellulase genes in the Δ*Mttrc-1* mutant grown on Avicel. In contrast, there were no differences in the transcription of genes encoding cellulase regulators (e.g., CLR-1, CLR-2, and CLR-4) between the Δ*Mttrc-1* mutant and the MtWT strain. This interesting finding led us to test whether MtTRC-1 can directly regulate cellulase gene transcription, similar to some classic cellulase regulators (e.g., CLR-1/2 and XYR-1) (21, 22). Notably, like other typical regulators of cellulase gene transcription, our EMSA, DNase I footprinting, and ChIP-qPCR results indicated that MtTRC-1 can directly regulate *Mtcbh-1* expression by binding to the promoter region *in vitro* and *in vivo* (Fig. 5–7). Our findings imply that MtTRC-1 directly regulates cellulase production at the transcriptional level (Fig. 8).

In summary, MtTRC-1 is demonstrated to be a major cellulase regulator in the thermophilic fungus *M. thermophila*. A possible model was proposed for the distinctive regulatory roles of MtTRC-1 as illustrated in Fig. 8. More specifically, MtTRC-1 can directly regulate cellulase gene *Mtcbh-1* expression, while also modulating the transcription level of the UPR key factor *Mthac-1* (Fig. 8). MtHAC-1-dependent UPR pathway is an essential factor for the cellulase secretion capacity of *M. thermophila*. These observations indicate the mechanisms regulating cellulase production form two levels network involving the HAC-1 mediated UPR signaling, as well as the transcriptional regulation of the major cellulase genes in parallel. The conservation of MtTRC-1 in filamentous fungi suggests this mechanism might be conserved, making it potentially relevant for engineering filamentous fungi to produce large amounts of industrially useful proteins.

## MATERIALS AND METHODS

### Strains and growth conditions.

*M. thermophila* strain ATCC 42464 was obtained from the American Type Culture Collection (ATCC). Escherichia coli DH5α and BL21 cells, which were used for vector construction/propagation and gene expression, respectively, were cultured in Luria–Bertani broth containing kanamycin or ampicillin (100 μg mL^−1^). The *M. thermophila* strains were grown as slant cultures on Vogel’s minimal medium (MM) supplemented with 2% glucose at 45°C, respectively, for 10 days to obtain conidia. Regarding the flask cultures, 10-day-old conidia from the *M. thermophila* strains were added to 100 mL liquid medium containing 1× Vogel’s salts and 2% (wt/vol) Avicel or cellobiose (final concentration, 1 × 10^6^ conidia mL^−1^). The *M. thermophila* cultures were incubated at 45°C with shaking at 150 rpm under constant light for 5 days, respectively, unless stated otherwise. For the media shift experiments, cultures were first grown for 16 h on 100 mL 1× Vogel’s salts medium supplemented with 2% (wt/vol) glucose, after which the mycelia were collected and washed five times in 1× Vogel’s salts medium. The mycelia were transferred to 100 mL fresh 1× Vogel’s salts medium containing 2% (wt/vol) Avicel or soluble starch and incubation was continued for the indicated time periods. For fungal hyphal growth assays, conidia were diluted to 1 × 10^6^ conidia mL^−1^ in 0.02% Tween 80 solution. An equal volume of the solution (1 μL) was inoculated onto the center of the MM plates containing various carbon sources (1%) and growth continued for 4 days at 45°C.

### Phylogenetic analysis.

Putative orthologs of Mycth_90344 in selected fungal species were identified on the basis of the best reciprocal BLAST hits during searches of the National Center for Biotechnology Institute (NCBI) protein database (*E* value < 10^−5^ applied as a cutoff). A phylogenetic tree was then constructed according to the maximum likelihood method, with 1,000 bootstrap replicates, using the MEGA 6 program.

### Construction of *M. thermophila ΔMycth_90344* and *ΔMthac-1* strains using the CRISPR-Cas9 system.

All primer sequences used in this study are listed in Table S1. The deletion of *Mycth_90344* mediated by our CRISPR-Cas9 system was performed as previously described (15). The guide RNA (gRNA) targeting *Mycth_90344* or *Mthac-1*, including the synthetic gRNA scaffold sequence and the target DNA sequence in *MtTRC-1* (5′-GCGTACACGTACGGGAGTTC*cgg*-3′; PAM is italicized) or *Mthac-1* (5′-GGCGTTAACACATACCACCT*tgg*-3′; PAM is italicized), was under the control of the *M. thermophila* U6 promoter. The gRNA expression cassettes for *Mttrc-1* and *Mthac-1* were constructed via overlapping PCR and cloned into the pJET1.2/blunt cloning vector, which yielded the corresponding plasmids U6p-Mycth_90344-sgRNA and U6p-Mthac-1-sgRNA, respectively. To construct the donor DNA sequences, the 5′ and 3′ flanking fragments of *Mycth_90344* (597 bp/579 bp) and *Mthac-1* (614 bp/616 bp) were separately amplified by PCR using *M. thermophila* ATCC 42464 wild-type (MtWT) strain genomic DNA as the template. The selectable marker cassette P*trpC*-*neo* was amplified by PCR using the p0380-neo plasmid as the template (14). The 5′ and 3′ fragments and P*trpC*-*neo* were assembled and inserted into pUC118 using the Gibson Assembly Cloning Kit (New England Biolabs, Ipswich, MA, USA) to generate donor-*Mycth_90344* and donor-*Mthac-1*. For the subsequent target gene deletion, total 10 μg PCR products of the gRNA expression PCR cassette U6p-target-sgRNA (1.0 μg), donor DNA of target gene (3.0 μg), and the Cas9-expression PCR cassette Ptef1-Cas9-TtprC (6.0 μg) were mixed at the same molar concentration ratio and cotransformed into protoplasts of the *M. thermophila* wild-type strain MtWT. Transformation of *M. thermophila* protoplasts was performed according to a previously described procedure (44). The transformants were screened on the basis of neomycin resistance using 80 μg mL^−1^ G418 after a 4-day culture. They were then confirmed by PCR analysis.

### Complementation and overexpression of *Mycth_90344* in *M. thermophila*.

To generate complementation strains, the upstream region (2,375 bp), the downstream region (751 bp), and the full-length *Mycth_90344* and NCU10006 sequences (1,438 bp and 951 bp, respectively) were amplified from MtWT and NcWT, respectively, using specific primer pairs and then ligated into the pAN52-bar plasmid at the BglII and XbaI sites (45) using the Gibson kit to generate the complementation plasmids pAN52-bar-Mycth_90344 and pAN52-bar-NCU10006. The *ΔMycth_90344* deletion mutant was transformed with the complementation plasmids via PEG-mediated protoplast transformation (44). To overexpress Mycth_90344 under the control of the strong constitutive *tef1* (MYCTH_2298136) promoter of *M. thermophila*, the MtWT *Mycth_90344* coding region (1,377 bp) was amplified by PCR and inserted into pAN52-P*tef1*-T*trpC*-*bar* at the SpeI and BamHI sites. The constructed recombinant plasmid was used to transform MtWT protoplasts. Colonies were grown for 4 days on selective medium and screened for resistance to 100 μg mL^−1^ phosphinothricin (indicative of *bar* expression). The presence of the transgenes was confirmed by PCR. For intracellular localization analyses of Mycth_90344, the *gfp* gene amplified from the pPK2BarGFPD plasmid (14) and the PCR-amplified MtWT *Mycth_90344* coding region without the stop codon were assembled and incorporated into pAN52-P*tef1*-T*trpC*-*bar*. To overexpress Mycth_90344-GFP under the control of its native promoter region, the amplified *Mycth_90344* full-length region (3,810 bp) and the *gfp* gene were assembled and inserted into pAN52-*bar* at the BglII and BamHI sites. The resulting pAN52-P*tef1*-*Mttrc-1-gfp*-T*trpC*-*bar* and pAN52-P*Mttrc-1*-*Mttrc-1-gfp*-T*trpC*-*bar* recombinant plasmid were inserted into the MtWT strain via protoplast transformation. Colonies grown for 4 days were screened according to phosphinothricin resistance (100 μg mL^−1^). Transformants were verified by a PCR analysis and the detection of GFP fluorescence in conidia.

### Secreted protein and enzyme activity analyses and dry weight measurement.

The total extracellular protein content in culture supernatants was determined using the Bio-Rad DC Protein assay kit (Bio-Rad, Hercules, CA, USA). A 30-μL aliquot of unconcentrated culture supernatant was loaded onto a polyacrylamide gel (Novex NuPAGE precast protein gels; Thermo Fisher Scientific, Waltham, MA, USA) to examine the proteins by SDS-PAGE. Endoglucanase and endo-1,4-β-xylanase activities in the culture supernatants were determined using the Azo-CM-Cellulose assay kit (Megazyme, Wicklow, Ireland) and the Azo-Xylan kit (Megazyme, Wicklow, Ireland), respectively. Exoglucanase activity was assayed as described by Deshpande et al. (65) with slight modifications. Briefly, the enzyme activity was measured at 50°C using 1.0 mg mL^−1^ *p*-nitrophenyl-β-d-cellobioside (Sigma-Aldrich, St. Louis, MO, USA) as the substrate in 50 mM citrate buffer (pH 4.8) containing 1 mg mL^−1^ d-glucono-1,5-σ-lactone. The reaction mixture containing 250 μL enzyme and 250 μL 1.0 mg mL^−1^ substrate in 50 mM citrate buffer (pH 4.8) was incubated for 10 min at 50°C, after which the reaction was terminated by adding 500 μL 1 M Na_2_CO_3_. The release of *p*-nitrophenol (*p*NP) was measured by monitoring the change in absorbance at 420 nm. The control contained inactive enzyme boiled at 100°C for 10 min. A standard curve was prepared using *p*NP. For the exoglucanase activity analysis, 1 U enzymatic activity was defined as the amount of *p*NP released from the substrate by 1 mL enzyme in 1 min under standard assay conditions.

The mycelial biomass dry weight of the Avicel cultures was measured by degrading the mycelia and weighing the residual solid (Avicel) as previously described (66). The mycelia was degraded by boiling in a water bath for 2 h of the acetic nitric reagent as described by Updegraff (66). The acetic nitric reagent is prepared by mixing 150 mL 80% acetic acid and 15 mL concentrated nitric acid. The mycelial biomass dry weights of the Avicel cultures were estimated at 5 days postinoculation. At the end of the time course experiments, the fungal biomass was harvested, dried, and weighed. The relative proportion of mycelia to residual Avicel was calculated by solubilizing the fungal biomass in boiling acetic nitric reagent and then washing the residual mass thoroughly before drying and reweighing. The mycelial dry weight was defined as the dry weight of the original culture minus that of the reaction mixture.

### Subcellular localization of MtTRC-1-GFP in *M. thermophila*.

To localize GFP fusion proteins via microscopy, the *M. thermophila* strains in liquid MM medium were grown for 16 h at 45°C. The hyphae were harvested, washed several times with Vogel’s salts medium, transferred to inducing medium containing 2% (wt/vol) Avicel or glucose, and then grown for another 24 h. Before imaging, the hyphae were incubated with 1 mg mL^−1^ DAPI for 15 min. The hyphae were examined using the Olympus BX51 fluorescence microscopy system and images were processed using ImageJ software.

### Analysis of *Mttrc-1*-specific transcriptomes.

Ten-day-old conidia from the *M. thermophila* strains were collected and used to inoculate 1× Vogel’s salts medium containing 2% glucose. Following a 16-h incubation at 45°C, the fungal mycelia were collected and washed four times with 1× Vogel’s salts medium and then transferred to fresh medium containing 2% (wt/vol) Avicel or soluble starch as the carbon source. The incubation was continued for the indicated time periods. After culturing, the mycelia of each strain were harvested by vacuum filtration and immediately ground to a fine powder in a pestle and mortar with liquid nitrogen for the subsequent extraction of total RNA. The total RNA was extracted by using the TRIzol reagent (Invitrogen, Carlsbad, CA, USA). Total RNA samples for each strain were purified using the Qiagen RNeasy minikit with digested DNase I (Qiagen, Hilden, Germany).

RNA sequencing (RNA-seq), reads mapping, and quantification were accomplished according to the method described previously by Li et al. (12). The purified RNA samples, with RNA integrity number > 8.0 determined using an Agilent 2100 Bioanalyzer (Agilent Technologies, Palo Alto, CA, USA), were sequenced by Novogene Corporation (Tianjin, China), who constructed cDNA libraries and sequenced using the Illumina Novaseq 6000 platform to generate 150-bp paired-end reads. All RNA-seq data in this study were generated by sequencing three independent biological replicates. The clean reads were mapped to the predicted transcripts for the *M. thermophila* ATCC 42464 genome (7) with < 2-base mismatching, using TopHat (v2.0.12) (67). Raw counts of reads that uniquely mapped to only one gene were calculated for each gene by HTSeq-count (v0.13.5) (68) and used for normalizing transcript abundance (reads per kilobase per million mapped reads [RPKM]) and analyzing differential gene expression using the DEseq2 package (v1.30.1) (69). The criteria used to identify genes that were significantly differentially expressed between growth conditions were as follows: expression level fold change > 2.0 (log_2_ ratio >1 or < −1) and DESeq adjusted *P* < 0.05.

### Analysis of *Mthac-1* mRNA unconventional splicing.

The unconventional splicing of *Mthac-1* was analyzed as previously described, with some modifications (57). The MtWT cultures pregrown for 16 h were shifted to fresh medium containing 2% glucose or 2% Avicel with 0 to 10 mM DTT to induce the UPR pathway for 4 h at 45°C. The mycelia were harvested by vacuum filtration and immediately homogenized in liquid nitrogen for the subsequent extraction of total RNA. Total RNA from frozen samples was isolated with TRIzol reagent (Invitrogen, Carlsbad, CA, USA) and further purified using the Qiagen RNeasy minikit with digested DNase I (Qiagen, Hilden, Germany). Total RNA was then reversely transcribed in cDNAs using the iScript cDNA Synthesis Kit (TOYOBO, Osaka, Japan) according to the manufacturers’ instructions. The *Mthac-1* coding region was amplified by PCR and inserted into the pJET1.2/blunt cloning vector for sequencing. The high MW band was indicative of the conventional splicing of *Mthac-1* (unspliced *Mthac-1*). The unconventional splicing of *Mthac-1* (spliced *Mthac-1*) was reflected by the lower MW bands.

### Quantitative real-time PCR.

For the RT-qPCR analysis, *M. thermophila* strains were grown for 16 h at 45°C in 1× Vogel’s salts medium supplemented with 2% (wt/vol) glucose. The mycelia of each strain were collected and washed several times with 1× Vogel’s salts medium and then cultured for incubation in fresh medium containing 2% (wt/vol) glucose, Avicel, or soluble starch as the carbon source. The incubation was continued for the indicated time periods. To simulate ER stress, 10 mM DTT (final concentration) was added to the glucose medium of the *M. thermophila* cultures pregrown for 16 h. The mycelia were cultured for another 4 h before being harvested by vacuum filtration and immediately ground to a fine powder in a pestle and mortar with liquid nitrogen for the subsequent extraction of total RNA. Total RNA from frozen samples was isolated with TRIzol reagent (Invitrogen, Carlsbad, CA, USA) and further purified using the Qiagen RNeasy minikit with digested DNase I (Qiagen, Hilden, Germany). The qPCR analysis was performed using the iScript cDNA Synthesis Kit, the SYBR green Realtime PCR Master Mix (TOYOBO, Osaka, Japan), and the CFX96 real-time PCR detection system (Bio-Rad, Hercules, CA, USA). Each reaction was completed in triplicate. The actin gene (MYCTH_2314852) was used as an endogenous control for all experiments. The transcript level of each gene was estimated using the 2^–ΔΔCt^ method (70). The ratio of the transcript level in the mutant to that in the MtWT control was calculated as the relative transcript level.

### Reactive oxygen species analysis.

Reactive oxygen species production was measured using the fluorescent probe DCFH-DA (Sigma-Aldrich, St. Louis, MO, USA) as previously described (71). The *M. thermophila* strains were grown in 100 mL minimal medium (10^6^ conidia mL^−1^) at 45°C for 6 h with shaking (150 rpm) and then treated with 20 μg mL^−1^ DCFH-DA for another 30 min. The cultures were collected and washed three times with phosphate-buffered saline (PBS) buffer before being transferred to PBS buffer containing 10 mM DTT and then incubated for 30 min. Samples were harvested, washed, and resuspended in PBS buffer and then analyzed using the SpectraMax M2e microplate reader (Molecular Devices, USA).

### Expression and purification of the MtTRC-1 DNA-binding domain.

The sequence encoding the MtTRC-1 DBD (amino acids 355 to 450) was amplified by PCR using MtWT cDNA. The accuracy of the amplified fragment was confirmed by sequencing. The PCR fragment was inserted into the pGEX-4T-1 plasmid (GE Healthcare, Piscataway, NJ, USA) at the BamHI and XhoI sites to generate the pGEX-MtTRC-1-DBD recombinant plasmid, which was subsequently introduced into E. coli BL21(DE3) cells for protein expression. The GST-tagged proteins were purified and verified as previously described (45).

### Electrophoretic mobility shift assays.

Different Cy5-labeled DNA fragments were used as probes in the gel-shift experiments according to the methods by Chen et al. (72). For the MtTRC-1-binding experiments, the Cy5-labeled *Mtcbh-1* (Mycth_109566) promoter region (P1, −297 to −1; P2, −559 to −242; P3, −834 to −505; P4, −1,106 to −780; P5, −1,381 to −1,052; P6, −1,640 to −1,327; P7, −1,843 to −1,572) and the Cy5-labeled *Mthac-1* (Mycth_2310995) promoter region (P1, −353 to −1; P2, −641 to −291; P3, −820 to −532; P4, −1,052 to −712; P5, −1,288 to −914; P6, −1,572 to −1,203; P7, −1,898 to −1,473) were amplified by PCR amplification using MtWT genomic DNA as the template. The PCR products were purified by electrophoresis and quantified using the NanoDrop 2000c spectrophotometer (Thermo Fisher Scientific, Waltham, MA, USA). The subsequent binding experiments were performed using a modified gel mobility shift assay as previously described (72). Briefly, in each EMSA reaction, different quantities of recombinant proteins were incubated with a constant amount (10 ng) of the Cy5-labeled DNA probes (individually) at 25°C for 30 min in buffer containing 0.2 mg mL^−1^ fish sperm DNA (Sangon, Shanghai, China), 20 mM Tris–base (pH 7.5), 2 mM DTT, 10 mM MgCl_2_, 20 mM KCl, 0.5 μg μL^−1^ calf bovine serum albumin (BSA), and 5% glycerol. In competition experiments to verify the binding specificity, unlabeled DNA probes were added to the reaction in 100-fold excess over the labeled probes. After incubation, protein-bound and free DNA were separated by electrophoresis on nondenaturing 5% (wt/vol) polyacrylamide gels with 0.5 × TBE running buffer (89 mM Tris-base, 89 mM boric acid, 1 mM EDTA, pH 8.0) at 100 V for 1 h at 4°C. Finally, gels were visualized by the Tanon-5200 Chemiluminescent Imaging System (Tanon Science and Technology, Shanghai, China). The experiments were performed at least three times to achieve stability.

### DNase I footprinting assay and site-directed mutagenesis.

The DNase I footprinting assays were performed as previously described (45). Briefly, PMtcbh1-FP (−909 to −618) and PMthac1-FP (−1,898 to −1,675) were incorporated into the pJET1.2/blunt cloning vector to, respectively, generate pJET-PMtcbh1-FP and pJET-PMthac1-FP, which were used as the targets. To prepare fluorescent FAM-labeled probes, the *Mtcbh1* and *Mthac1* promoter regions in the pJET-PMtcbh1-FP and pJET-PMthac1-FP plasmids, respectively, were amplified by PCR using the 2× TOLO HIFI DNA polymerase premix (TOLO Biotech, Shanghai, China) and primers pJET1.2-F2 (FAM) and pJET1.2-R2. The FAM-labeled probes were purified and quantified. For each assay, 400-ng probes were incubated with different amounts of purified MtTRC-1 in a total volume of 40 μL. After a 30-min incubation at 25°C, 10 μL solution comprising 0.015 U DNase I (Promega, Madison, WI, USA) and 100 nmol freshly prepared CaCl_2_ was added. The reaction mixture was incubated at 37°C for 1 min. The reaction was stopped by adding 140 μL DNase I stop solution (200 mM unbuffered sodium acetate, 30 mM EDTA, and 0.15% SDS). Samples were first treated with phenol-chloroform, after which the extracts were precipitated with ethanol. Pellets were dissolved in 30 μL MilliQ water. The preparation of the DNA ladder, electrophoresis, and data analysis were performed as previously described (45). The assays were repeated three times to ensure the results were consistent.

To decrease the likelihood of altering the DNA structure during the site-directed mutagenesis, the base mutations were as follows: G to A and C to T (37). The mutated probes were synthesized by Life Science Research Services Company (Genewiz, Suzhou, China).

### Generation of MtTRC-1 antiserum.

The method used to generate MtTRC-1 antiserum was similar to that described by Lin et al. (73). Briefly, the pGEX-4T-1 vector and E. coli BL21(DE3) cells were used to express the GST-MtTRC-1 (amino acids 1 to 181) fusion protein. The purified recombinant protein was used as the antigen for immunizing rabbits, which generated rabbit polyclonal antiserum as previously reported (73).

### Chromatin immunoprecipitation analysis.

The ChIP-qPCR assays were performed according to a previously described protocol (34). Briefly, the fungal mycelia were fixed in 100 mL Avicel medium containing 1% formaldehyde at 45°C for 15 min with shaking before the cross-linking was quenched by adding 4 mL 2.5 M glycine and incubating for 5 min. The mycelia were then collected, ground in liquid nitrogen, and lysed in buffer consisting of 50 mM HEPES, pH 7.5, 137 mM NaCl, 1 mM EDTA, 1.0% Triton X-100, 0.1% sodium deoxycholate, 0.1% SDS, 1 mM PMSF, 1 μg mL^−1^ leupeptin, and 1 μg mL^−1^ pepstatin A. The crude lysate was sonicated to obtain an average DNA fragment size of 500 bp. The immunoprecipitation step was performed by adding 12.5 μL anti-MtTRC-1 antibody and then incubating at 4°C for 6 h. Additionally, 40 μL protein A/G beads precoated with 10 mg mL^−1^ BSA and 10 mg mL^−1^ fish sperm DNA were used for each immunoprecipitation. After the postimmunoprecipitation sequential washes, the DNA was eluted at 65°C for 4 h using elution buffer (1% SDS and 100 mM NaHCO_3_) and recovered by a proteinase K treatment at 45°C for 1 h, a phenol–chloroform extraction, and ethanol precipitation. The ChIP-qPCR analysis was performed using the precipitated chromatin DNA, the SYBR green Realtime PCR Master Mix, and the CFX96 real-time PCR detection system. Relative DNA enrichment was calculated as a percentage of the input DNA. All experiments were performed at least three times.

### Statistical significance tests.

All assays were repeated three times to generate data. The significance of any differences was determined via a one-way ANOVA. For all tests, *P* < 0.05 was set as the threshold for significance.

### Data availability.

All of the data generated in this study are available and included in the main text and supplemental material. The RNA-seq raw data are available in the Gene Expression Omnibus database (accession number GSE205182) at the National Center for Biotechnology Information (NCBI).
