# Supplementary material for: MtTRC-1, a Novel Transcription Factor, Regulates Cellulase Production via Directly Modulating the Genes Expression of the Mthac-1 and Mtcbh-1 in Myceliophthora thermophila
Source: Appl Environ Microbiol. 2022 Sep 27;88(19):e01263-22. doi: 10.1128/aem.01263-22 (PMC9552611; doi:10.1128/aem.01263-22)
Supplement: Supplemental file 1 — Fig. S1 to S7. Download aem.01263-22-s0001.pdf, PDF file, 1.4 MB [file aem.01263-22-s0001.pdf]

Supplemental material for Applied and Environmental Microbiology

MtTRC-1, a novel transcription factor, regulates cellulase production via directly modulating the genes expression of the *Mthac-1* and *Mtcbh-1* in *Myceliophthora thermophila*

Nan Li[a,b,c,]*, Yin Liu[a,b,c], Defei Liu[b,c], Dandan Liu[b,c,d], Chenyang Zhang[b,c], Liangcai Lin[a], Zhijian Zhu[b,c], Huiyan Li[a], Yujie Dai[a], Xingji Wang[e], Qian Liu[b,c,]*, Chaoguang Tian[b,c,]*

[a]College of Biotechnology, Tianjin University of Science & Technology, Tianjin 300457, China

[b]Key Laboratory of Systems Microbial Biotechnology, Tianjin Institute of Industrial Biotechnology, Chinese Academy of Sciences, Tianjin 300308, China

[c]National Technology Innovation Center of Synthetic Biology, Tianjin 300308, China

[d]State Key Laboratory of Agrobiotechnology and MOA Key Laboratory of Soil Microbiology, College of Biological Sciences, China Agricultural University, Beijing 100193, China

[e]Longda Biotechnology Inc, Shandong, Linyi 276400, China

*Address correspondence to Nan Li, lin@tib.cas.cn, Qian Liu, liu_q1@tib.cas.cn and Chaoguang Tian, tian_cg@tib.cas.cn

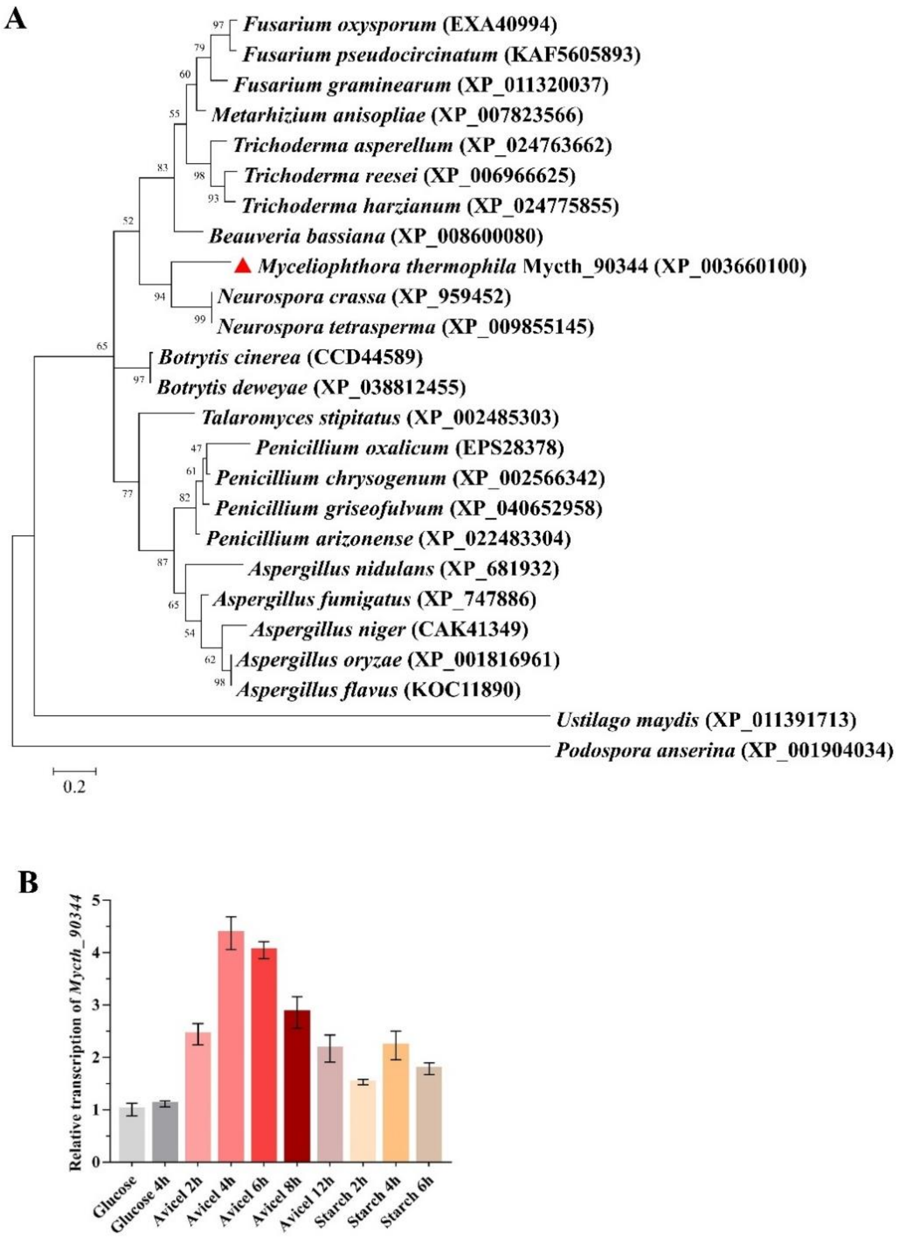

Fig. S1 Phylogenetic analysis of Mycth_90344 protein and the *Mycth_90344* transcription on different carbon sources. (A) Phylogenetic analysis of Mycth_90344 protein. Amino acid sequences of all proteins were obtained from NCBI database. Phylogenetic tree was constructed using MEGA 6.0 software with the neighbor-joining method. (B) The transcription of *Mycth_90344* on glucose, Avicel, and soluble starch was analyzed by RT-qPCR. Relative gene expression levels at the indicated time intervals were calculated and compared with that on glucose, which was set to 1.

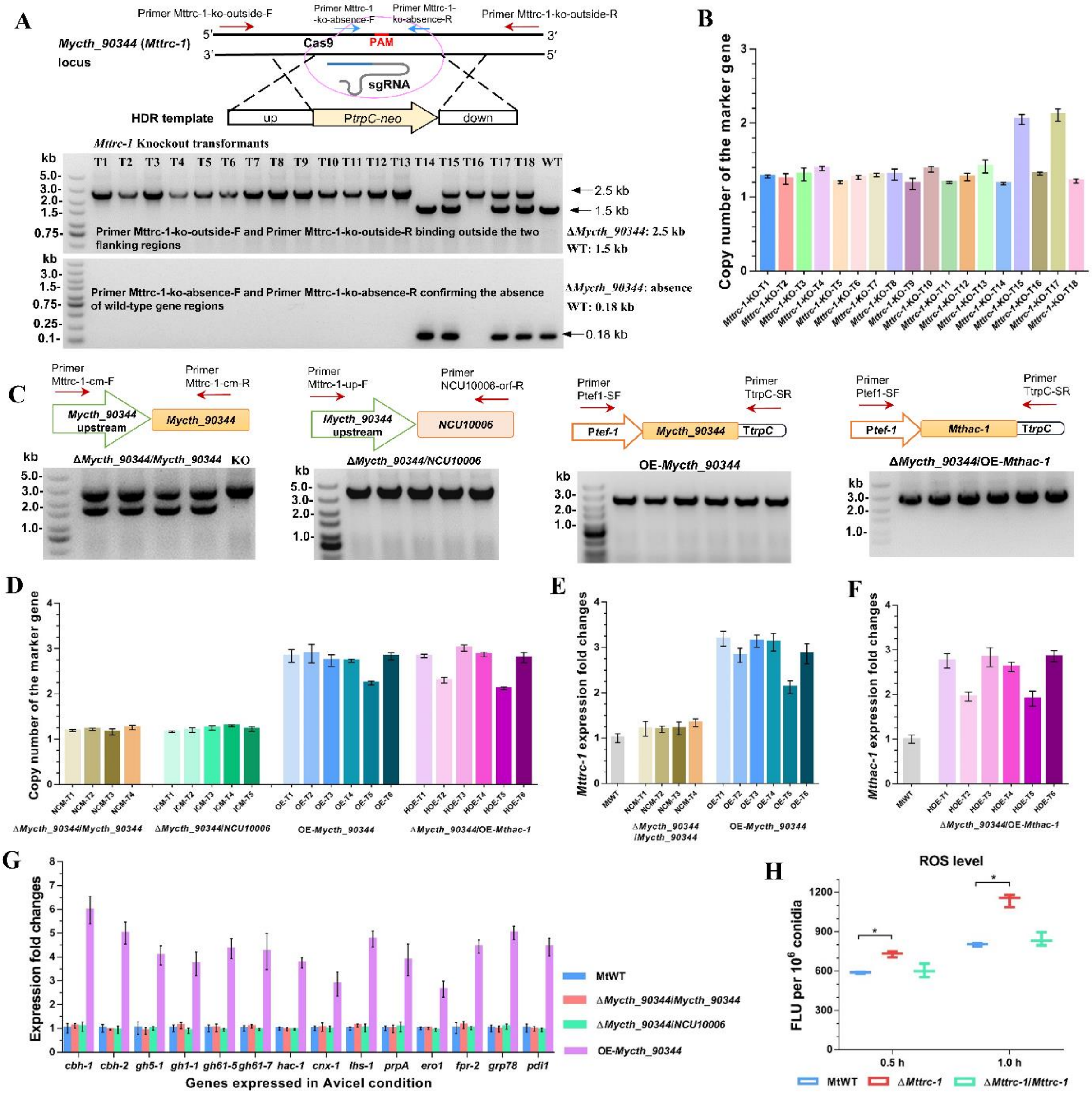

Fig. S2. Construction and verification of *M. thermophila* mutants Δ*Mycth_90344*, Δ*Mycth_90344*/*Mycth_90344* and Δ*Mycth_90344*/*NCU10006* and OE-*Mycth_90344* and Δ*Mycth_90344*/OE-*Mthac-1*. (A) Schematic view of genomic manipulation of the *Mycth_90344* (*Mttrc-1*) by using our CRISPR–Cas9 system and identification of the mutants via PCR with two paired primers. The expected length of disrupted transformants of *Mycth_90344* was 2.5 kb with primers 1 and 2 binding outside the flanking, while that of the MtWT (rightmost lane) was 1.5 kb. The absence of wild-type

gene length of the *Mycth_90344* disrupted transformants was confirmed with primers 3 and 4, while that of the MtWT (rightmost lane) was 0.18 kb. (B) Determination of the copy number of the maker gene in the *Mycth_90344*disrupted transformants by RT-qPCR analysis. (C) PCR verification of the complemented transformants Δ*Mycth_90344*/*Mycth_90344* and Δ*Mycth_90344*/*NCU10006*, and overexpressing strains OE-*Mycth_90344* and Δ*Mycth_90344*/OE-*Mthac-1*. (D) Determination of the copy number of the maker gene in the transformants of Δ*Mycth_90344*/*Mycth_90344*, Δ*Mycth_90344*/*NCU10006*, OE-*Mycth_90344* and Δ*Mycth_90344*/OE-*Mthac-1* by RT-qPCR analysis. (E) The transcription level of *Mttrc-1* in the MtWT, Δ*Mycth_90344*/*Mycth_90344* and OE-*Mycth_90344* strains by RT-qPCR. Strains were pre-grown in MM-glucose for 16 h, washed and transferred to 2% (w/v) Avicel medium for 4 h incubation. (F) The transcription level of *Mthac-1* in the MtWT and Δ*Mycth_90344*/OE-*Mthac-1* strains by RT-qPCR. Strains were pre-grown in MM-glucose for 16 h, washed and transferred to 2% Avicel medium for 4 h induction. (G) RT-qPCR analyses of major cellulase genes and essential genes involved in ER stress in the MtWT, Δ*Mycth_90344*/*Mycth_90344*, Δ*Mycth_90344*/*NCU10006*, and OE-*Mycth_90344* strains. (H) The ROS level was determined after treating fungal mycelia with 1 mM DTT for 0.5 and 1 h. *$P < 0.05$. Error bars represent the SD from three replicates.

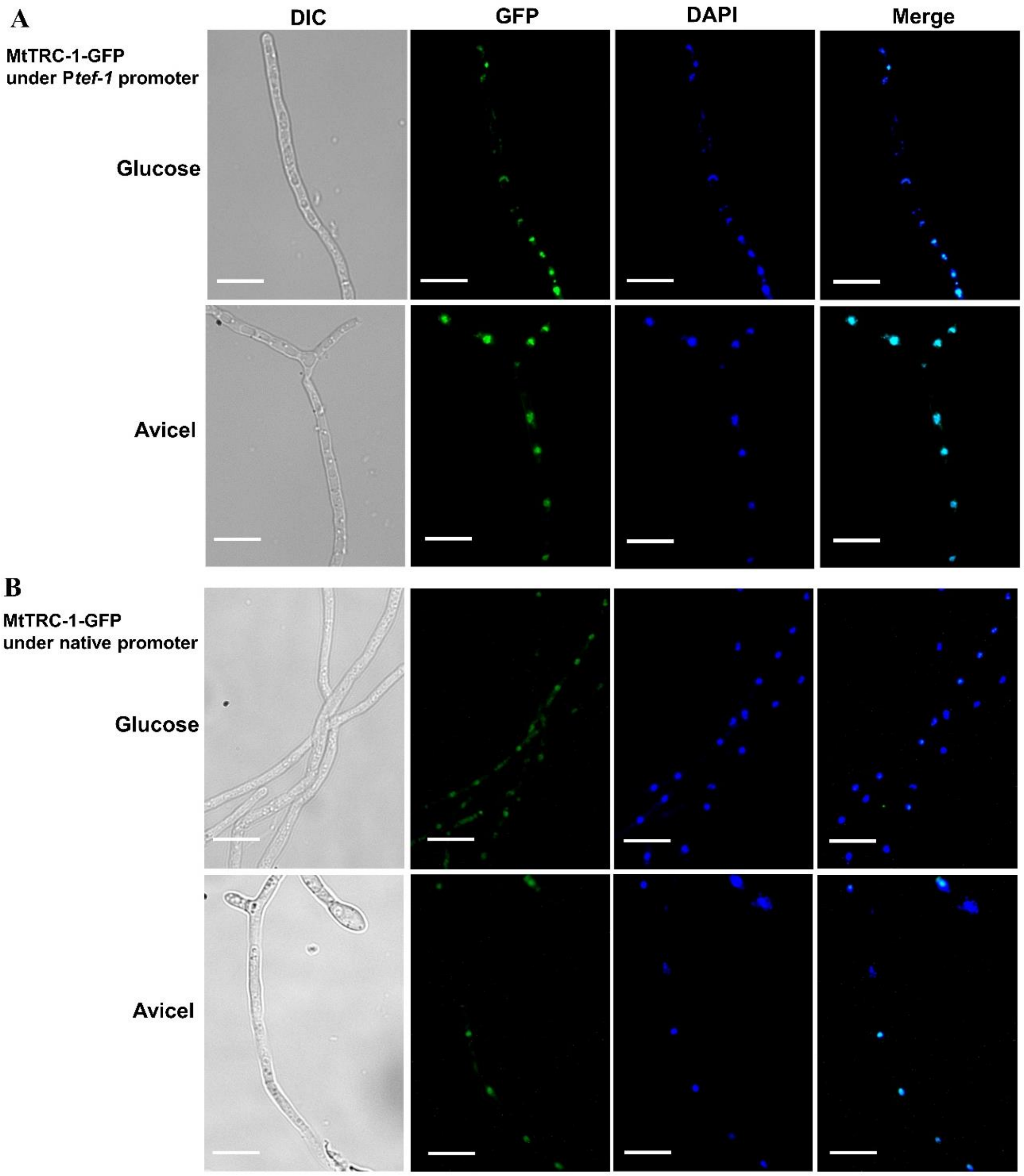

Fig. S3. Subcellular location of MtTRC-1 in *M. thermophila.* Strains expressing the MtTRC-1-GFP fusion protein under the strong promoter Ptef-1 (A) and the native promoter region (B) were pre-grown for 16 h in minimal medium supplemented with 2% (w/v) sucrose as the sole carbon source and then transferred to medium containing 2% (w/v) glucose or 2% (w/v) Avicel for a 24-h incubation. The localization of MtTRC-1 was monitored by recording the EGFP signal. Nuclei were stained by DAPI. Samples

were examined using the TCS SP5 II laser scanning confocal microscope (Leica). Scale bars = 10 μm.

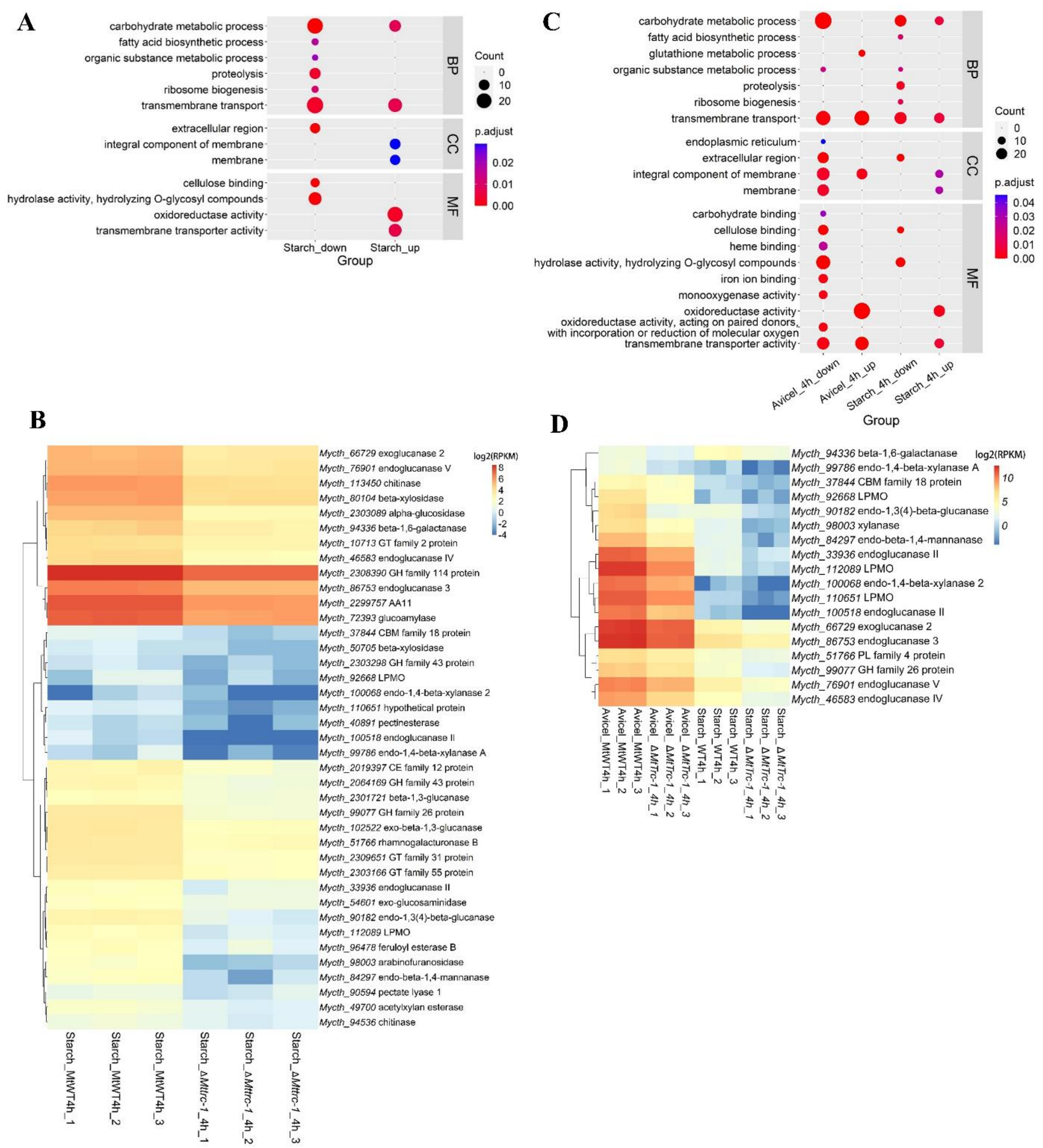

Fig. S4. Comparative transcriptomics analysis of Δ*Mttrc-1* and MtWT grown in soluble starch medium for 4 h. (A) Gene Ontology analysis of the down-regulated genes and up-regulated genes differentially expressed between Δ*Mttrc-1* and MtWT on starch medium. (B) Heatmap analysis of expression profiles for the CAZy genes with statistically significant differences in transcript levels between Δ*Mttrc-1* vs. MtWT

under starch condition. Log-transformed expression values are color-coded. (C) Gene Ontology analysis of the down-regulated genes and up-regulated genes differentially expressed between Δ*Mttrc-1* and MtWT on both Avicel and starch medium. (D) Heatmap analysis of expression profiles for the CAZy genes with statistically significant differences in transcript levels between Δ*Mttrc-1* vs. MtWT under both Avicel and starch condition. Log-transformed expression values are color-coded.

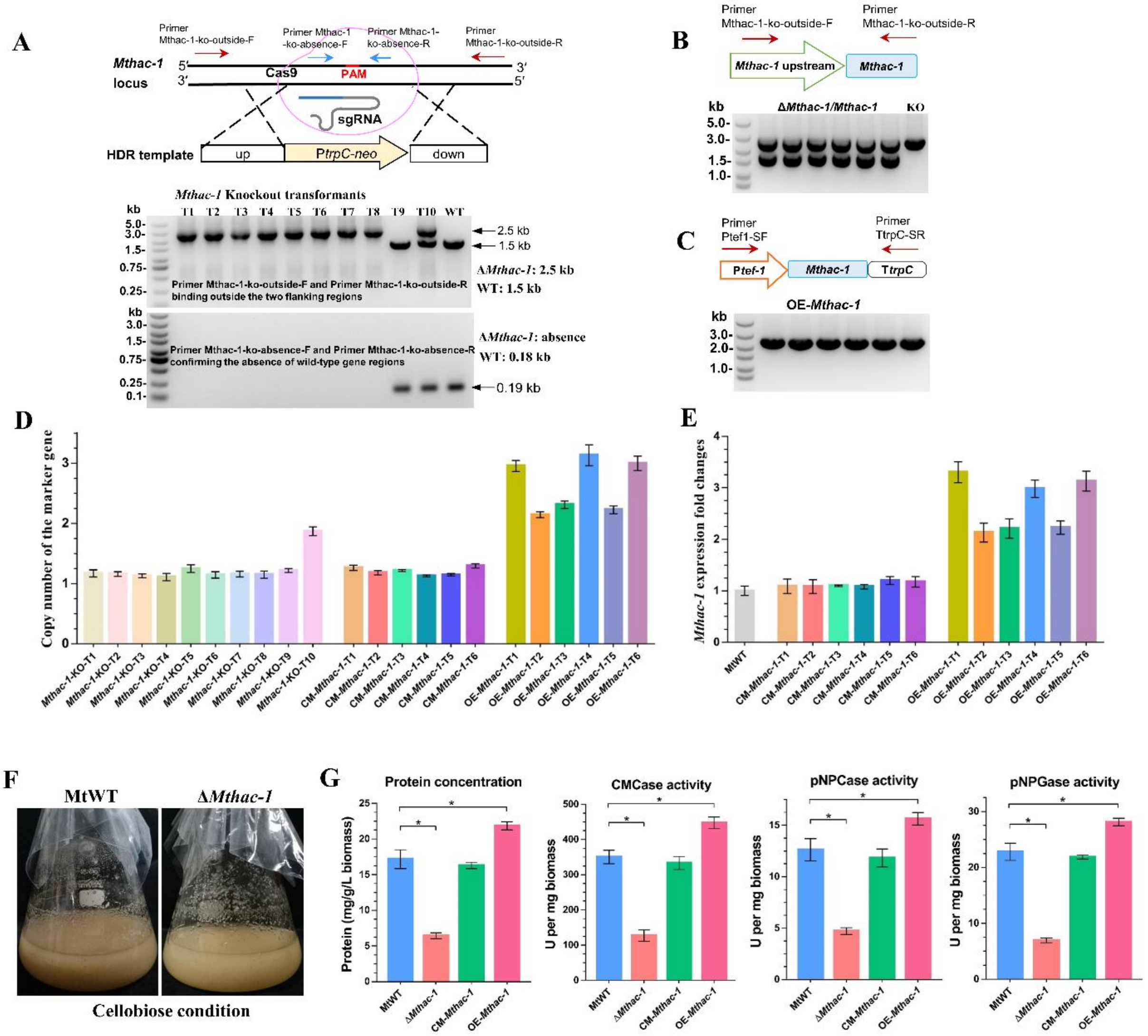

Fig. S5. Construction and verification of *M. thermophila* mutants Δ*Mthac-1*, Δ*Mthac-1*/*Mthac-1* and OE-*Mthac-1*. (A) Schematic view of genomic manipulation of the *Mthac-1* by CRISPR–Cas9 system and identification of their mutants via PCR with two paired primers. The expected length of the *Mthac-1*disrupted transformants was 2.5 kb with primers 1 and 2 binding outside the flanking, while that of the MtWT (rightmost lane) was 1.5 kb. The absence of wild-type gene length of the *Mthac-1* disrupted transformants was confirmed with primers 3 and 4, while that of the MtWT (rightmost lane) was 0.19 kb. (B-C) PCR verification of the complemented transformantsΔ*Mthac-*

1/*Mthac-1* (CM-*Mthac-1*) and overexpressing strains OE-*Mthac-1*. (D) Determination of the copy number of the maker gene in the transformants of Δ*Mthac-1*, Δ*Mthac-1*/*Mthac-1* (CM-*Mthac-1*) and OE-*Mthac-1* by RT-qPCR analysis. (E) The transcription level of *Mthac-1* in the MtWT, Δ*Mthac-1*/*Mthac-1* (CM-*Mthac-1*) and OE-*Mthac-1* strains by RT-qPCR. Strains were pre-grown in MM-glucose for 16 h, washed and transferred to 2% Avicel medium for 4 h induction. (F) Growth of MtWT and Δ*Mthac-1* on cellobiose medium after 4 days. (G) Assays for protein concentration and enzyme activities of the Δ*Mthac-1* and MtWT after 4 days culture in cellobiose medium.

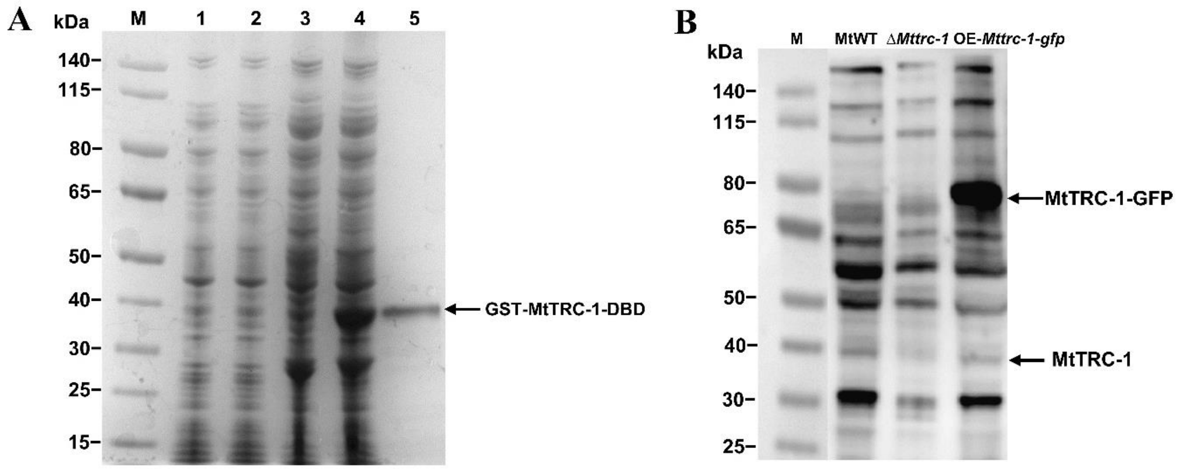

Fig. S6. SDS-PAGE results showing purified GST tag fused MtTRC-1 DNA binding domains (GST-MtTRC-1-DBD indicated by the arrow) (A). Lane M is the protein marker. Lane 1, proteins produced in BL21 (DE3) before 1.0 mM IPTG incubation; Lane 2, unpurified GST- MtTRC-1-DBD before 1.0 mM IPTG incubation; Lane 3, proteins produced in BL21 (DE3) after IPTG incubation; Lane 4, unpurified GST-MtTRC-1-DBD after IPTG incubation; Lane 5, purified GST-MtTRC-1-DBD. (B) Western blot analyses showing that polyclonal antibody specifically recognizes MtTRC-1 protein in MtWT and OE-*Mttrc-1-gfp* strains but not in Δ*Mttrc-1* mutant. Arrow indicated MtTRC-1 protein band detected by MtTRC-1 polyclonal antibody.

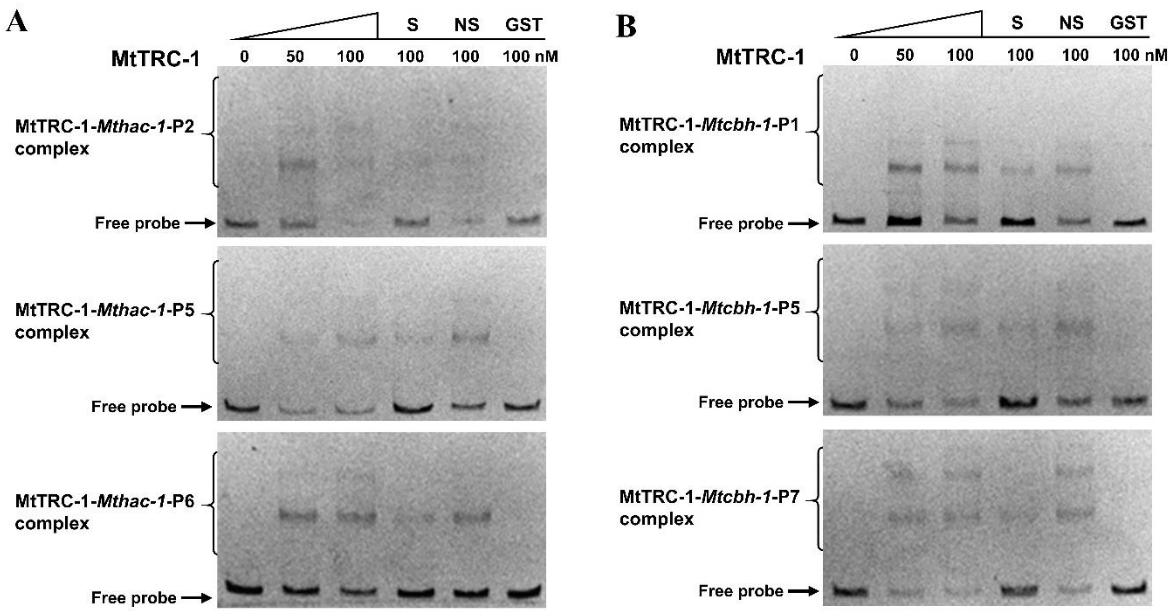

Fig. S7. Electrophoretic mobility shift assays (EMSAs) of DNA binding of MtTRC-1to upstream regions of *Mthac-1* (A) and *Mtcbh-1* (B). Each lane contained 10 ng Cy5-labeled probe and indicated amounts of purified MtTRC-1 binding domain (nM). The shifts were verified to be specific by adding 100-fold excess of unlabeled specific (S) and nonspecific (NS) competitor DNA. Purified GST was used as a negative control to exclude nonspecific binding.
